# ﻿Species delimitation, biogeography, and natural history of dwarf funnel web spiders (Mygalomorphae, Hexurellidae, *Hexurella*) from the United States / Mexico borderlands

**DOI:** 10.3897/zookeys.1167.103463

**Published:** 2023-06-14

**Authors:** Rodrigo Monjaraz-Ruedas, Raymond Wyatt Mendez, Marshal Hedin

**Affiliations:** 1 Department of Biology, San Diego State University, San Diego, California 92182–4614, USA San Diego State University San Diego United States of America; 2 2002 W Pogo Hill, Portal AZ 85632 Unaffiliated Portal United States of America

**Keywords:** Biogeography, micro-endemism, Mojave Desert, sky islands, taxonomy, ultraconserved elements

## Abstract

The rarely encountered spider genus *Hexurella* Gertsch & Platnick, 1979 includes some of the smallest mygalomorph spiders in the world, with four poorly known taxa from central and southeastern montane Arizona, southern California, and northern Baja California Norte. At time of description the genus was known from fewer than 20 individuals, with sparse natural history information suggesting a vagrant, web-building, litter-dwelling natural history. Here the first published taxonomic and natural history information for this taxon is provided in more than 50 years, working from extensive new geographic sampling, consideration of male and female morphology, and sequence capture-based nuclear phylogenomics and mitogenomics. Several new species are easily diagnosed based on distinctive male morphologies, while a complex of populations from central and northern Arizona required an integrative combination of genomic algorithmic species delimitation analyses and morphological study. Four new species are described, including *H.ephedra***sp. nov.**, *H.uwiiltil***sp. nov.**, *H.xerica***sp. nov.**, and *H.zas***sp. nov.** Females of *H.encina* Gertsch & Platnick, 1979 are also described for the first time. It is predicted that additional new species will ultimately be found in the mountains of central and northwestern Arizona, northern mainland Mexico, and the Mojave Desert of California.

## ﻿Introduction

**Figure 1.**
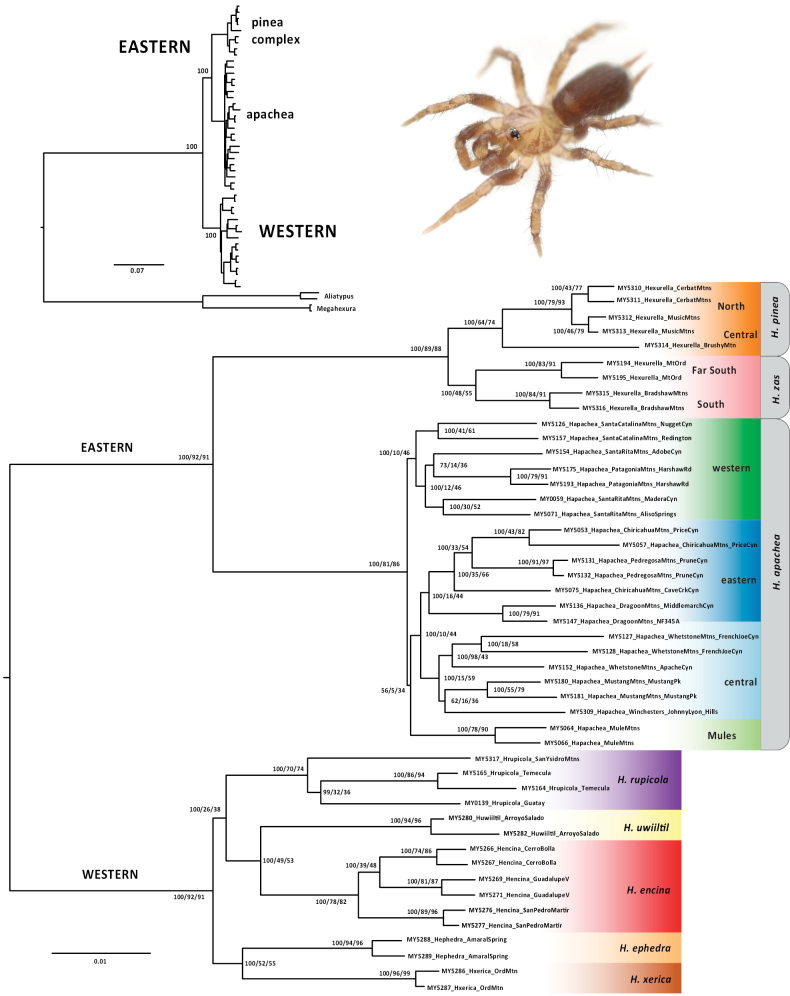
Ingroup-only UCE concatenated ML tree. Specimen numbers correspond to those in Suppl. material 1. Nodal values correspond to bootstrap / gCF / sCF (gCF and sCF values rounded to nearest integer). Inset upper left – Rooted ML tree with bootstrap values shown for primary lineages (see also Suppl. material 4). Inset upper right – adult male *Hexurellarupicola* (San Diego County, CA).

Dwarf funnel web spiders in the genus *Hexurella* Gertsch & Platnick, 1979 are infrequently encountered and poorly known. In the first and only revision of this taxon Gertsch and Platnick (1979) described four new species in this newly erected genus. This included two species from central and southeastern montane Arizona, one species from far southern California, and one species from northern Baja California Norte. These mygalomorphs were found to be very small as adults, ranging in size from 2.5–5 mm, placing them among the smallest mygalomorph spiders in the world (Fig. 1 inset). Gertsch and Platnick (1979) described the genus as uncommon, with two species known only from their respective type localities, and the other species known from two or three sampling locations each. At the time of description, the genus was known from fewer than 20 individuals, with sparse natural history information suggesting a vagrant, web-building, cryptic (in litter or under rocks) natural history.

In a phylogenomic analysis of all atypoid mygalomorph genera, Hedin et al. (2019) sampled two *Hexurella* species and found these to occupy a distinct phylogenetic branch sister to all remaining atypoids, well separated from other described genera of the original family Mecicobothriidae. These authors dismantled Mecicobothriidae based on these results and described a new monogeneric family (Hexurellidae) to house the phylogenetically relictual *Hexurella*. The lineage leading to this genus was estimated to have diverged from other atypoids approximately 300 million years ago (confidence intervals ranging from 250–354 Mya), while the common ancestor of the sampled species from Arizona and California was estimated to have lived approximately 60 million years ago (21–185). While this latter timeframe is likely a strong overestimate, the available phylogenomic data suggests a relatively deep history for stem (and possibly crown) groups of this relictual lineage of miniature mygalomorphs.

The biogeographic histories of animal taxa that include disjunct representatives in upland habitats of southern California / Baja California Norte and central and southeastern Arizona are mostly unstudied. One emphasis has been on the disjunct habitats in which such animals occur, which are dominated by sclerophyllous woody plant taxa. One hypothesis is that these plant communities, and perhaps the animals dependent upon them, are part of an Oligocene-Miocene Madro-Tertiary Geoflora (Raven and Axelrod 1978; Lancaster and Kay 2013; Baldwin 2014). DiDomenico and Hedin (2016) tested the MTG hypothesis in phalangodid harvesters of the *Sitalcinasura* species group. These harvesters are like *Hexurella* in distribution and habitat preference (and sometimes found together), although *Sitalcina* Banks, 1911 are generally more moisture dependent. DiDomenico and Hedin (2016) found a timing consistent with MTG origins and surprisingly found that southern California desert canyon taxa are sister to central Arizona upland taxa, rather than to geographically adjacent southern California Mediterranean taxa. Similar patterns are possible in *Hexurella*, with other possibilities including reciprocal east <> west monophyly (clades separated by the Colorado River), or perhaps directional paraphyly (e.g., AZ taxa nested within a western clade or vice versa). More phylogenetic studies are needed to understand comparative biogeographic patterns in upland taxa of this region.

The Sky Islands of southern Arizona and northern Mexico are a biologically diverse region where mountain ranges, isolated by arid lowland basins, act as refugia for various montane lineages (summarized in Moore et al. 2013). Spanning the gap between the Sierra Madre Occidental and the Rocky Mountains, the floral and faunal communities of this area are a mixture of northern and southern elements, varying gradually by latitude and steeply by elevation. Studies on arachnids here have shown relatively deep genetic divergences between populations, implying histories older than the last glacial maximum when oak forest connected the mountains (Masta 2000; Bryson et al. 2013a, b; Hendrixson et al. 2015; Derkarabetian et al. 2016). *Hexurellaapachea* Gertsch & Platnick, 1979 is found in many of these ranges, and their strict microhabitat preferences and potentially poor dispersal capabilities make them an interesting subject for studies of sky island biogeography.

The revisionary research presented here is based on original and recent collections of *Hexurella* populations from Arizona, southwestern California, and northwestern Baja California Norte. This sampling has greatly increased the number of known populations for the genus and includes detailed natural history information. New distributional records extend further south into Baja California Norte, further north into the Mojave Desert of California, into the mountains of northwest Arizona, and include extensive records for the montane sky islands of southeastern Arizona. For these new specimens we have studied male and female morphology, and for a representative sample have gathered ultraconserved element (UCE) nuclear phylogenomic data, and mitogenomic data as UCE by-catch. We also conduct formal genetic species delimitation analyses, using the newly developed methods SPEEDEMON (Douglas and Bouckaert 2022) and DELINEATE (Sukumaran et al. 2021). A combination of analyses and multiple lines of evidence allow us to revise the genus, describe new species, and better understand biogeographic patterns. We herein describe four new *Hexurella* species, including *H.ephedra* sp. nov., *H.uwiiltil* sp. nov., *H.xerica* sp. nov., and *H.zas* sp. nov. We also describe previously unknown females for *H.encina* Gertsch & Platnick, 1979. Several *Hexurella* species are highly geographically localized, deserving conservation attention and future monitoring as modern species representing a phylogenetically ancient lineage.

## ﻿Materials and methods

### ﻿Specimen and geographic sampling

We collected specimens from Arizona, southern California, and Baja California Norte, on the traditional and ancestral lands of the Yuhaaviatam/Maarenga’yam (Serrano), Kumeyaay, Paipai (Akwa’ala), Ko’lew (Kiliwa), Hualapai, Tohono O’odham, Yavapai, Western Apache, and Chiricahua Apache peoples. We searched appropriate microhabitats for spiders and collected specimens by hand or using an aspirator. Most spiders were preserved in the field in either 80% EtOH for morphological study, or 100% EtOH for DNA analysis. Specimens preserved for DNA analysis were later stored in a -80 °C freezer. Geographic location data were taken in the field using a cell phone and later verified using ACME Mapper (https://mapper.acme.com/).

If immatures (**imm**) were collected in association with adults from the same geographic location and in the same microhabitats, these specimens were attributed to the species found at that location (Suppl. material 1), reflecting a lack of known sympatry in the genus. Some locations represented only by immatures were included in phylogenomic analyses, with post hoc identifications based on species clade membership.

### ﻿UCE data collection and processing

We gathered UCE data for 51 specimens, including 47 *Hexurella*, and a handful of distant atypoid outgroups (two *Megahexurafulva* (Chamberlin, 1919), *Aliatypusgulosus* Coyle, 1975, and *Aliatypusisolatus* Coyle, 1975; Suppl. material 1). We sampled from type localities (or near type localities) for the four previously described *Hexurella* species. Genomic DNA was extracted from leg tissues using the DNeasy Kit (Qiagen GmbH, Hilden, Germany), with at least 250 ng of genomic DNA used for UCE library preparation. Library preparation was performed both at SDSU and at RAPID Genomics. SDSU experiments followed standard methods as previously used for arachnids (Starrett et al. 2017). Target enrichment was performed using the myBaits UCE Spider 2Kv1 kit (Arbor Biosciences; Kulkarni et al. 2020), with libraries sequenced using 150 bp, paired-end Illumina HiSeq 4000 sequencing at the DNA Technologies Core, UC Davis, CA, USA. For the remaining experiments genomic DNA was sent to RAPID Genomics for library preparation (using the Spider 2Kv1 probe set) and Illumina sequencing.

Bioinformatic analyses were carried out on the Mesxuuyan HPC at SDSU. Raw demultiplexed reads were quality-filtered and cleaned of adapter contamination with Trimmomatic (Bolger et al. 2014; parameters: PE ILLUMINACLIP:$adaptersfasta:2:30:10:2:keepBothReads LEADING:5 TRAILING:15 SLIDINGWINDOW:4:15 MINLEN:40). Cleaned reads were assembled into contigs using SPADES v. 3.13.0 (Prjibelski et al. 2020) with --sc and --careful options. To remove contigs with low coverage and/or depth we followed the correction workflow implemented in PHYLUCE v. 1.7.1 (Faircloth 2016; https://phyluce.readthedocs.io/en/latest/daily-use/daily-use-4-workflows.html#correction). After contig filtering we used PHYLUCE to map and identify UCE loci, mapping contigs against merged arachnid and spider probe sets (see Maddison et al. 2020) using default (80, 80) matching values. Individual loci were aligned using MAFFT (Katoh et al. 2009) and trimmed using Gblocks (Castresana 2000) using parameters: b1: 0.50, b2: 0.70, b3: 10, b4: 4. After generating UCE alignments we performed additional data filtering, including ‘per sample’ and ‘per alignment’ filtering. For ‘per sample’ filtering, we removed samples with low numbers of UCEs (phyluce_align_get_taxon_locus_counts_in_alignments), removed highly divergent sequences using CIAlign (Tumescheit et al. 2022), and removed sequences shorter than 80% of the total alignment length using a custom python script (fasta_drop.py, Suppl. material 2). For ‘per alignment’ filtering, we filtered by completeness using PHYLUCE to generate 80% occupancy matrices.

### ﻿UCE alignments and SNPs

We created two datasets for phylogenomic analyses, including ingroup + outgroup (51 samples, 588 loci), and ingroup only (47 samples, 608 loci) (Suppl. material 1). We conducted a maximum likelihood (ML) analysis with IQ–TREE (Minh et al. 2020a) using individual UCE loci alignments as separate partitions, with 1000 replicates of ultrafast bootstrapping and optimal model search using ModelFinder (Kalyaanamoorthy et al. 2017). We also reconstructed an ingroup-only concatenated ML tree in IQ–TREE and used gene trees from individual UCE alignments to calculate gene (gCF) and site (sCF) concordance factors (Minh et al. 2020b).

An ingroup-only species tree was estimated under a multispecies coalescent model using ASTRAL v. 5.7.7 (Mirarab et al. 2014; Mirarab and Warnow 2015; Rabiee et al. 2019). Input gene trees for ASTRAL were estimated using IQ–TREE 2 with 1000 replicates of ultrafast bootstrapping and treated as unrooted. Zhang et al. (2018) suggest that collapsing nodes with low support values for individual gene trees improves accuracy in ASTRAL; we used newick utilities (‘nw_ed’) to collapse branches with bootstrap values below 50. Internal branch lengths for ASTRAL analyses were estimated in coalescent units, with branch support measured as both quartet scores and local posterior probability values (a function of number of loci and quartet frequencies; Sayyari and Mirarab 2016).

For SNP-based analyses (see below) we extracted SNPs from ingroup-only UCE alignments using the script ’snps_from_uce_alignments’ (Andermann et al. 2019). We allowed for missing data (‘--include_missing’) and called one random SNP per locus. We also excluded samples with more than 70% missing data (MY5057, MY5127, MY5128; Suppl. material 1), resulting in 608 SNPs for 44 taxa.

### ﻿Mitochondrial data collection and analysis

Because no mitochondrial reference is available for *Hexurella* we followed a two-step workflow for gathering approximately full mitogenome UCE by-catch data for ingroup samples. This workflow included mitochondrial contig identification and genes recovery. We first mapped assembled UCE contigs to a reference mitogenome of *Megahexurafulva* (unpublished data) using MitoFinder v. 1.4.1 (Allio et al. 2020). Due to the high divergence between *Hexurella* and *Megahexura*, we were not able to find all mitochondrial genes using only MitoFinder. We proceeded by using the recovered MitoFinder contigs to manually edit and annotate mitochondrial genes using Geneious Prime 2022 to generate a final *Hexurella* reference. Then, using custom scripts (‘mtdna_alignments.sh’, Suppl. material 2) we mapped clean raw reads against the *Hexurella* reference created in the previous step using BWA (Li 2013). Resulting BAM files were used as input for calling consensus sequences using SAMTOOLS v. 1.15 (Danecek et al. 2021). Lastly, consensus sequences were merged, aligned, and trimmed using MAFFT (Katoh et al. 2009) and Gblocks (Castresana 2000) as implemented in PHYLUCE 1.7.1 (Faircloth 2016).

Phylogenetic analysis of a concatenated mitochondrial matrix was conducted using IQ–TREE ML searches. This matrix was partitioned by gene with a best–fitting partition scheme found by possibly merging partitions; support was measured using 1000 replicates of ultrafast bootstrap.

### ﻿Divergence time estimation

Divergence times were estimated using BEAST v. 2.4.8 (Bouckaert et al. 2014) using the mitochondrial COI data only (recovered for 46 *Hexurella* samples; Suppl. material 3). We explored three different unpartitioned COI clock rates, including a “standard” arthropod mitochondrial rate of 2.3%/ Ma (0.0115 substitutions/site/myr) (Brower 1994), a COI rate of 2.5%/ Ma (0.0125 substitutions/site/myr) proposed for spiders (Bidegaray-Batista and Arnedo 2011), and a COI rate of 3.36%/ Ma (0.0169 substitutions/site/myr) proposed for insects (Papadopoulou et al. 2010). We specified a GTR model without partitions as suggested by Modelfinder and used a relaxed lognormal clock with a Birth-Death model as a tree prior, with a gamma distribution (Alpha = 2.0, Beta = 2.0 in BEAUti). The ‘ucld.mean’ prior varied to reflect COI rate (see above); for all runs we specified a Log normal distribution with a standard deviation of 0.07 and a ‘ucld.Stdev’ prior with a gamma distribution (Alpha = 0.5396, Beta = 0.3819 in BEAUti). The input tree topology was constrained to that recovered with the full concatenated (15 genes) mitogenomic tree. For each rate, two runs of 1 × 10^7 generations were specified and merged in LogCombiner v. 2.4.8 (Bouckaert et al. 2014), with the initial 25% discarded as burn-in. Convergence of chains and effective sample sizes were assessed using Tracer v. 1.7.1 (Rambaut et al. 2018). Finally, trees were summarized using a maximum clade credibility tree in TreeAnnotator v. 2.4.8 (Bouckaert et al. 2014).

We used MEGA v. 11 (Tamura et al. 2021) to calculate between-group mean Kimura 2-parameter COI distances (K2-P; Kimura 1980), using recovered phylogenomic clades and subclades to define groups.

### ﻿Species delimitation

We approached the species delimitation problem in two different ways. First, we used an integrative, morphology-first approach, where a priori morphological species were “validated” via phylogenomic analyses. Here we defined species as “single populations (= collection localities) or sets of populations that share diagnostic male morphologies, strongly supported (using multiple measures) by nuclear phylogenomic monophyly.”

For most *Hexurella* taxa this integrative approach appeared to perform well, with clear morphological groupings with minimal intraspecific morphological variation, strongly supported by nuclear phylogenomics (see Results). One exception involved the *H.pinea* “complex” in central and northwestern Arizona. Here, five separate sample locations included males that differed to various degrees from each other. These five populations together formed a phylogenomic clade, itself with divergent internal branches (see Results). Here, the distinction between intraspecific variation vs. interspecific divergence (i.e., species boundaries) was less clear.

To address this uncertainty, we used formal genomic algorithmic species delimitation analyses. We used two alternative approaches, SPEEDEMON (Douglas and Bouckaert 2022) and DELINEATE (Sukumaran et al. 2021). Both methods incorporate prior information about species boundaries for informing species limits in the group of interest, including for “unknown” specimens / populations. This prior information can come from different external sources, including morphology, natural history, previous phylogenetic analyses, etc., and is incorporated as a type of “prior” in parameter exploration (Sukumaran et al. 2021).

SPEEDEMON (Douglas and Bouckaert 2022) uses a birth-death collapse model implemented in the BEAST package (Bouckaert et al. 2014). This method allows for joint estimation of species boundaries and phylogeny without limits to the number of individuals and species included (albeit with an increase in computational time) and includes a user defined threshold (epsilon, *ε*) for decisions of species limits (Douglas and Bouckaert 2022). An advantage of SPEEDEMON over other multispecies coalescent methods like BPP (Yang and Rannala 2010) or STACEY (Jones 2016) is its flexibility in the use of alignments or SNPs (Douglas and Bouckaert 2022), allowing the incorporation of larger data sets with reasonable computational times. A potential disadvantage of this method is that epsilon values have an important influence on the number of species recovered, although epsilon can be informed by external knowledge by considering expected divergence between known species or by performing sensitivity analyses with different epsilon values (Douglas and Bouckaert 2022).

SPEEDEMON analyses were performed in BEAST v. 2.6.7 (Bouckaert et al. 2014) using unlinked SNPs in SNAPPER (Stoltz et al. 2021). Samples were assigned a priori to species based on morphological groupings validated with phylogenomic data. For the *H.pinea* complex samples were assigned to four geographic (and phylogenomic) lineages, including North, Central, South, and FarSouth (see Results). Yule and collapse weight priors were set as default; for the SNAPPER coalescent rate a gamma distribution prior was set with a mean of 46, following the SNAPPER manual (https://github.com/BEAST2-Dev/beast-docs/releases/download/v1.0/snapper-delimitation-tutorial-2021.zip) after calculating the expected maximum tree height. Two values were explored for the epsilon threshold. First, we calculated the divergence between “known” morphological species in units of substitutions per site by calculating patristic distances using the concatenated IQ-TREE analysis. We used the minimum observed interspecific divergence between “known” species (in this case, *H.encina* and *H.uwiiltil* sp. nov.) of ~0.04 and set epsilon to one half this value (*ε* = 0.02; see Douglas and Bouckaert 2022). We ran a second analysis using the same parameters as above but used *maximum observed intraspecific* patristic distances (~ 0.037) to set epsilon (*ε* = 0.0185). Both SPEEDEMON analyses included two independent runs of 6x10^7 generations; runs were merged and 25% of samples were discarded as burn-in, with convergence assessed using Tracer v. 1.7.1 (Rambaut et al. 2018).

In addition to SPEEDEMON we also used DELINEATE (Sukumaran et al. 2021), which uses a “species completion rate” (SCR) to inform the process of species delimitation (Sukumaran et al. 2021). DELINEATE models evolution using a birth-death process and requires the use of an ultrametric MSC tree in which the tips of the tree represent either populations of a single species or independent species (Sukumaran et al. 2021). The SCR is then calculated using the same data and tree by providing a priori information on which populations in the tree represent species (if known) or by providing a previously calculated SCR if species are completely unknown (Sukumaran et al. 2021). The DELINEATE workflow (https://jeetsukumaran.github.io/delineate/index.html) calls for the estimation of a MSC tree as a first step, using either a BPP analysis and the delimitation result as population assignments or using StarBeast (Heled and Drummond 2010) if populations are known. We circumvented this step by summarizing trees resulting from SPEEDEMON, using a maximum clade credibility tree obtained with TreeAnnotator v. 2.4.8 (Bouckaert et al. 2014). We ran two independent DELINEATE analyses using different SPEEDEMON trees (from different *ε* values). Known and unknown species assignments were assigned in the same way as for SPEEDEMON, including four geographic lineages within the *H.pinea* complex as unknown. Following one of the SPEEDEMON results that lumped *H.ephedra* sp. nov. with *H.xerica* sp. nov. (see Results), we also treated *H.xerica* sp. nov. as unknown in DELINEATE analyses. Analyses were run by calling delineate-estimate partitions and reporting only partitions with a cumulative probability of 0.99.

### ﻿Taxonomy

Our descriptions of somatic and genitalic morphology paralleled the characters also emphasized by Gertsch and Platnick (1979). Appendage and palpal measurements were taken from the left appendage and are reported in mm. Measurements were taken using an eyepiece micrometer at 3–4× magnification with an Olympus SZX12 stereomicroscope fitted with 10× ocular lenses. Embolus and conductor characters were scored using an Olympus BX40 microscope; these characters are not readily visible in our lower magnification digital images (see below).

Specimens were digitally imaged using a Visionary Digital BK plus system including a Canon 40D digital camera and Infinity Optics Long Distance Microscope. Individual images were combined into a composite image using Helicon Focus v. 6.6.2 software, then edited using Adobe Photoshop. Images were taken with specimens immersed in filtered 70% EtOH, using KY jelly to secure samples.

Female spermathecae were dissected from specimens using fine forceps, immersed in BioQuip specimen clearing fluid on a depression slide, then imaged directly in this fluid on slides. We imaged spermathecal organs for most taxa for sake of completeness but did not emphasize these characters in our taxonomic diagnoses. Although Gertsch and Platnick (1979) commented on the potential informativeness of spermathecal characters, in hindsight, none of their diagnostic comparisons involved sister taxa. We have found spermathecal characters to be less informative in distinguishing sister taxa, but admittedly have not explored all sister species comparisons. Also, we have found adequate taxonomic resolution in the combination of male morphology and phylogenomics, so have not needed to fully explore the taxonomic utility of female variation. Gertsch and Platnick (1979) also had very small female sample sizes. Studies in other mygalomorph taxa have found important intraspecific variation in spermathecal morphology, including variation among females at single locations, and left to right asymmetry in single individuals (e.g., Leavitt et al. 2015; Li et al. 2022). To fully understand intraspecific variation we would need to conduct a larger number of somewhat challenging specimen preparations. These preparations are destructive to the specimens, do not always result in usable results, and as suggested above, have not been particularly informative where explored. We are not arguing that female characters might not have future utility in this group of spiders; rather, we simply do not emphasize this character system here.

Holotype and paratype specimens from California and Arizona have been deposited at the Bohart Museum of Entomology (**BME**) at UC Davis. Holotype and paratype specimens from Baja California Norte have been deposited at the Centro de Investigación Científica y de Educación Superior de Ensenada, Baja California, Mexico (**CICESE**). All other specimens referenced with San Diego State University Terrestrial Arthropod Collection (**SDSU_TAC**).

## ﻿Results and discussion

### ﻿Specimen and geographic sampling

The total morphological and molecular sample considered is summarized in Suppl. material 1. This included 200 adult *Hexurella* specimens (84 adult males) from 49 unique collecting events.

### ﻿UCE data and results

Raw UCE read data have been submitted to the Sequence Read Archive (BioProject ID: PRJNA95350). Final alignments were available for 51 samples and 588 loci (ingroup + outgroup), or for 47 samples and 608 loci (ingroup only). The ingroup-only matrix included a concatenated length of 364,758 base pairs. All input matrices, analysis log files, and output tree files can be found in Suppl. material 3.

**Figure 2.**
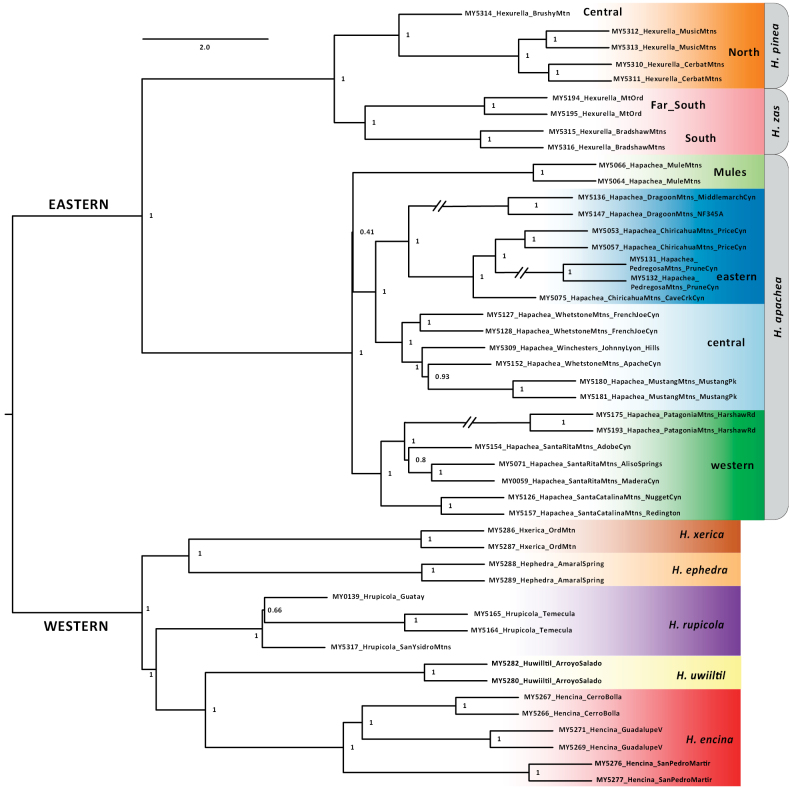
Ingroup-only UCE ASTRAL species tree. Specimen numbers correspond to those in Suppl. material 1. Included are local posterior probability values. Branch lengths in coalescent units for internal branches only, terminal branch lengths arbitrary.

**Figure 3.**
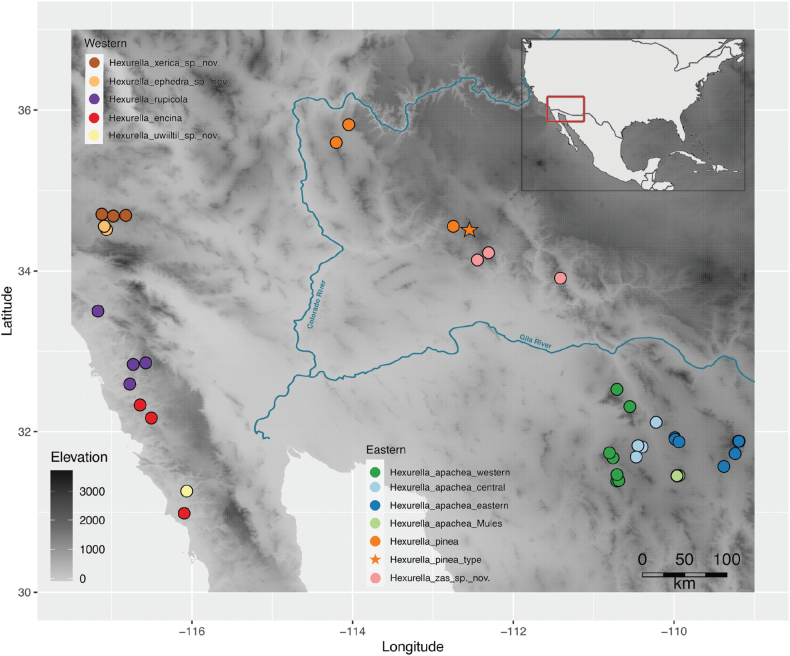
Map of all collection localities. Phylogeographic lineage names for *H.apachea* as in text. The unsampled type locality of *H.pinea* is designated with a special symbol; all other type (or near type) localities were sampled, see text.

Concatenated ML analyses including distant atypoid outgroups recovered a monophyletic *Hexurella*, with internal eastern (AZ) versus western (CA + Baja) clades (Fig. 1 inset, Suppl. material 4). This eastern versus western root placement was presumed in the ingroup-only concordance factor and ASTRAL analyses; very similar internal topologies are recovered in all three analyses (Figs 1, 2; Suppl. material 4). The western clade includes sister species in the Mojave Desert (*H.ephedra* sp. nov, *H.xerica* sp. nov.), sister to more southerly taxa (*H.rupicola* (*H.encina*, *H.uwiiltil* sp. nov.)); Figs 1–3). The eastern clade includes a *H.pinea* complex clade and a clade corresponding to *H.apachea*. The latter is subdivided into 4 distinct geographic subclades, including the Mule Mountains, western (e.g., Santa Catalinas, Santa Ritas), central (e.g., Whetstones, Mustangs), and eastern (e.g., Chiracahuas, Dragoons) montane subclades (Figs 1–3). Central plus eastern is well-supported in both concatenated and ASTRAL analyses, but central/eastern plus Mule Mountains is weakly supported in both (Figs 1, 2). As measured by bootstrap, concordance factors, or posterior probabilities, this is a rare weakly-supported node on these topologies.

### ﻿Mitochondrial data and results

Mitochondrial data for 47 *Hexurella* specimens were gathered as UCE by-catch (Suppl. material 1). The concatenated matrix included 9604 total sites, although not all genes were recovered for all specimens (10.5% missing data). Input matrices, analysis log files, and output tree files can be found in Suppl. material 3.

**Figure 4.**
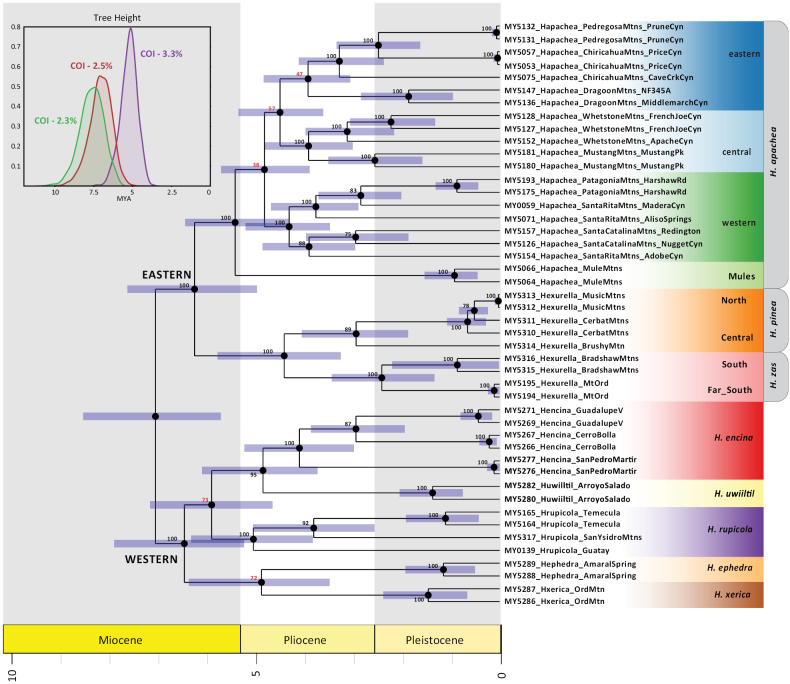
Mitochondrial COI time tree, based on 2.5% substitution rate analysis. Bootstrap values from whole mitogenome phylogenetic analysis (see Suppl. Material 5). Upper left inset: Density plot of tree root heights estimated using different COI substitution rate values.

Presuming an eastern versus western root placement, the mitochondrial topology is very similar to nuclear topologies (Fig. 4, Suppl. material 5). The western clade includes species in the Mojave (*H.ephedra* sp. nov., *H.xerica* sp. nov.) sister to more southerly taxa (*H.rupicola* (*H.encina*, *H.uwiiltil* sp. nov.)). The eastern clade includes the *H.pinea* complex sister to *H.apachea*, itself subdivided into 4 distinct geographic subclades, with subclade interrelationships uncertain because of weakly-supported nodes (Fig. 4, Suppl. material 5).

Between-group mean K2–P COI distances range from 10.5–12.7% in the western clade, 7.8–10.5% in the *H.pinea* complex, and 8.5–9.6% among geographic subclades within *H.apachea* (Table 1). As a point of comparison, K2–P COI distances range 10–13% for hypothesized cryptic species in the atypoid mygalomorph genus *Aliatypus* (Starrett et al. 2018). In a comparative analysis of multiple Australian mygalomorph genera, Castalanelli et al. (2014) used an uncorrected COI p-distance of 9.5% as a barcoding threshold. These authors found that 92% of “known” morphological species were congruent with molecular species boundaries at this threshold. In tarantulas, Hamilton et al. (2014) found a shallower COI barcode gap at ~ 5% divergence (uncorrected p-distance), with these genetic clusters corresponding to species boundaries based on multiple data types (e.g., morphology, behavior, geography).

**Table 1.** COI between-group mean K2–P distances. “*pinea_south*” and “*pinea_farSouth*” correspond to *H.zas* sp. nov.

	Mules	apachea_western	apachea_eastern	
**apachea_western**	0.096			
**apachea_eastern**	0.0947	0.0908		
**apachea_central**	0.0902	0.0886	0.0849	
	**pinea_north**	**pinea_central**	**pinea_south**	
**pinea_central**	0.0783			
**pinea_south**	0.0922	0.0795		
**pinea_farSouth**	0.1051	0.1022	0.078	
	**xerica**	**ephedra**	**rupicola**	**uwiiltil**
**ephedra**	0.1095			
**rupicola**	0.1133	0.1100		
**uwiiltil**	0.1115	0.1052	0.1104	
**encina**	0.1269	0.1186	0.1160	0.1077

### ﻿Biogeography

Nuclear and mitogenomic analyses recover well-supported eastern versus western clades (Figs 1, 2, 4), with a common ancestor diverging an estimated 5–7.5 million years ago (Fig. 4). An obvious hypothesis is that the Colorado River and/or the low desert habitats found on both sides of this river (Fig. 3) explain this primary divergence. The estimated age of the drainage of the Colorado River into the Gulf of California is ~ 4.1 Mya (Dorsey et al. 2007; Dolby et al. 2015); a subset of our clock estimate confidence intervals overlap with this timing.

The Colorado River barrier hypothesis is a general paradigm found in the literature, although as noted above, this pattern does not apply in phalangodid harvesters (DiDomenico and Hedin 2016). As detailed below in the Taxonomy section, our new collections have extended the known distribution of eastern taxa west towards the river (e.g., *H.pinea* in the Cerbat Mtns), and have extended the range of western taxa eastwards (new Mojave species), at least at higher latitudes (Fig. 3). Understanding if the river corridor is indeed acting as a barrier will require additional collections from specialized microhabitats in the challenging and isolated small ranges found both east and west of the river. Prior to this study *Hexurella* would have never been contemplated from such uninviting habitats. We hypothesize that other upland taxa with perceived western versus eastern disjunct distributions might also span this region where novel discoveries await.

Biogeographic patterns and estimated divergence times within *H.apachea* are discussed below in the Taxonomy section for this species.

### ﻿Species delimitation

We recognized six morphological species *a priori*, including the previously described *H.apachea*, *H.rupicola*, and *H.encina*, and three newly discovered morphologically distinctive species. These six morphological species are all also recovered with high support with nuclear phylogenomic data (as measured by ML bootstrap, concordance factors, ASTRAL local posterior probabilities; Figs 1, 2). Because of possible clinal variation in male morphology within the *H.pinea* complex (see below), we treated the five sampled populations as four separate geographic lineages and tested the species status of these lineages using genomic delimitation analyses.

SPEEDEMON *ε* = 0.02 recovers a seven species hypothesis with a support of 99.54%, with the *H.pinea* complex subdivided into two species (Table 2). This includes North + Central lineages together with a support of 99.99% (recovered together as a single species in 99.99% of topologies sampled) and South + FarSouth lineages together with a support of 99.94% (Table 2). Although we specified *H.ephedra* sp. nov. and *H.xerica* sp. nov. as distinct “known” species a priori, SPEEDEMON *ε* = 0.02 lumps these morphologically distinct taxa together as single species with a support of 99.6%. SPEEDEMON *ε* = 0.0185 recovers 10 distinct species, recognizing *pinea* North, Central, South, and FarSouth lineages as distinct species with high support (Table 2). *Hexurellaephedra* sp. nov. and *H.xerica* sp. nov. were likewise recovered as distinct species with 100% support each (Table 2). As expected, assigned values for SPEEDEMON epsilon values impacted the number of recovered *Hexurella* species (Douglas and Bouckaert 2022). The informed selection of epsilon using external data, as done here, can be helpful in delimiting “unknown” or conflicting lineages; choosing an appropriate epsilon in completely unknown groups will be more challenging.

**Table 2.** Results of species delimitation analyses, showing support for each species across different methods and parameters. Fixed = Lineage constrained as “known” in DELINEATE analyses. Merged cells denote lineages recovered as a single species. “*pinea complex* S” and “*pinea complex* FS” correspond to *H.zas* sp. nov.

Lineage	SPEEDEMON		DELINEATE	
	*ε* = 0.02	*ε* = 0.0185	*ε* = 0.02	*ε* = 0.0185
*H.apachea*	100	100	Fixed	Fixed
*H.encina*	100	100	Fixed	Fixed
*H.rupicola*	100	100	Fixed	Fixed
*H.uwiiltil*	99.64	100	Fixed	Fixed
*H.ephedra*	99.60	100	Fixed	Fixed
*H.xerica*		100	100	100
*H.pineacomplex* C	99.99	99.98	100	100
*H.pineacomplex* N		99.98	100	100
*H.pineacomplex* FS	99.94	100	100	100
*H.pineacomplex* S		100	100	100
**Total species**	**7**	**10**	**10**	**10**

DELINEATE recovers a 10 species hypothesis regardless of the different input SPEEDEMON trees used (from different *ε* values). Within the *H.pinea* complex, North, Central, South and FarSouth lineages are recovered as independent species each with a posterior support of 1 (Table 2). *Hexurellaephedra* sp. nov. and *H.xerica* sp. nov. are also recovered as distinct species with a posterior support of 1 (Table 2).

### ﻿Taxonomy

The taxonomy presented below is organized to follow phylogenomic results, including separate sections for eastern versus western lineages, and following sister taxon relationships within lineages. Fig. 3 shows the geographic distribution of sample locations, geographic lineages, and species.

#### Family Hexurellidae Hedin & Bond, 2019

##### ﻿Genus Hexurella

Taxon classificationAnimaliaAraneaeHexurellidae

Gertsch & Platnick, 1979

7F336E89-9BAD-5FAF-A834-53979E2B9946

###### Remarks.

We follow the generic diagnosis provided by Hedin and Bond in Hedin et al. (2019): adults males with a gently coiled embolus, posterior lateral spinnerets with four segments, and spermathecae composed of a single bursal opening branching into four or more elongate receptacles. As adults these spiders are also much smaller than other adult mygalomorphs from North America, except for the avicularioid *Microhexura* Crosby & Bishop, 1925. *Hexurella* differs from *Microhexura* in possessing abdominal tergites and six spinnerets.

#### Eastern lineage

Well-supported phylogenomic clade, currently known from east of the Colorado River in the uplands of northwestern, central, and southeastern Arizona.

**Included species.***Hexurellaapachea* Gertsch & Platnick, 1979, *Hexurellapinea* Gertsch & Platnick, 1979, *Hexurellazas* sp. nov.

##### ﻿ Hexurella apachea

Taxon classificationAnimaliaAraneaeHexurellidae

Gertsch & Platnick, 1979

BD232FB7-B147-5F07-83BE-720076519827

Figs 5 , 6

Hexurella apachea Gertsch and Platnick (1979): 29, figs 81, 83–85 (Dmf).

###### Material examined.

**Near-type locality material: USA – Arizona, Cochise Co.** • 1♂, 1 imm; Chiricahua Mtns., Cave Creek Canyon, 31.8815, -109.1978; 15 Mar. 2021; R.W. Mendez leg. – **Cochise Co.** • 1♂; Chiricahua Mtns., Cave Creek Canyon, 1 mi. E Southwest Research Station, FR 42, 31.8809, -109.1890; 12 Oct. 2021; R.W. Mendez leg.; RWM 21_050. – **Cochise Co.** • 5♂, 1♀, 2 imm; Chiricahua Mtns., Cave Creek Canyon, FR-42, Snowshed Trailhead, 31.8811, -109.1968; 20 Oct. 2021; R.W. Mendez, R.A. Mendez leg; RWM 21_057.

###### Non-type material.

***H.apachea* Eastern Lineage – Arizona, Cochise Co.** • 3♂, 4♀, 7 imm; Chiricahua Mtns., Price Canyon,31.7266, -109.2387; 16 Mar. 2021; R.W. Mendez leg. – **Cochise Co.** • 3♂, 6♀; Dragoon Mtns, 1 mi E West Stronghold trailhead, Cochise Trail 279, 31.9223, -109.9899; 30 Oct. 2021; R.W. Mendez, K. Silvestre-Bringas, E. Ciaccio leg.; RWM 21_061. – **Cochise Co.** • 5 imm; Dragoon Mtns, 2.8 mi up NF-345A, 0.4 mi down ravine, 31.9036, -109.9830; 21 Aug. 2021; R.W. Mendez, M.A. Leimroth leg.; RWM 21_027. – **Cochise Co.** • 3♂, 3♀, 2 imm; Dragoon Mtns, 2.8 mi up NF-345A, 31.8997, -109.9835; 17 Nov. 2021; R.W. Mendez, C.A. Hamilton leg.; RWM 21_076. – **Cochise Co.** • 1♀, 4 imm; Dragoon Mtns., Middlemarch Canyon, E of Middlemarch Pass, W of Pearce, 31.8729, -109.9399; 23–24 Jul. 2021; M. Hedin, R.W. Mendez leg.; MCH 21_084. – **Cochise Co.** • 1♂, 2♀, 4 imm; Pedregosa Mtns., Prune Canyon, 31.5668, -109.3800; 17 Apr. 2021; R.W. Mendez leg.

***H.apachea* Central Lineage – Cochise Co.** • 1♂, 1♀, 1 imm; SW of Winchester Mtns, Johnny Lyon Hills, W of Keith Peak, 32.1154, -110.2237; 17 Jan. 2022; R.W. Mendez, M.A. Leimroth leg.; RWM 22_009. – **Cochise Co.** • 3♀; Whetstone Mtns, French Joe Canyon, 31.8092, -110.3976; 23 Aug. 2021; R.W. Mendez leg. – **Cochise Co.** • 6♂, 3♀, 4 imm; Whetstone Mtns, French Joe Canyon, E French Joe Spring, 31.8107, -110.3945; 14 Nov. 2021; R.W. Mendez, C.A. Hamilton, M.A. Leimroth leg.; RWM 21_075. – **Pima Co.** • 4♀, 3 imm; Whetstone Mtns, 0.5 mi SE Willow Spring, Apache Canyon, 31.8193, -110.4571; 22 Aug. 2021; R.W. Mendez leg; RWM 21_030. – **Pima Co.** • 3♀, 3 imm; Whetstone Mtns, 0.75 mi E Willow Spring, Apache Canyon, 31.8252, -110.4487; 22 Aug. 2021; R.W. Mendez leg; RWM 21_029. – **Santa Cruz Co.** • 4♂, 8♀, 2 imm; Mustang Mtns, NW Mustang Peak, 31.6852, -110.4709; 14 Nov. 2021; R.W. Mendez, C.A. Hamilton, M.A. Leimroth leg.; RWM 21_074.

***H.apachea* Western Lineage – Pima Co.** • 2♂, 2♀, 8 imm; Santa Catalina Mtns, Redington Pass, 1 mi W Youtey Pasture Tank., Redington Road, 32.3107, -110.5508; 2 Oct. 2021; R.W. Mendez, D. Roth leg.; RWM 21_044. – **Pima Co.** • 1♀; Santa Catalina Mtns, Nugget Cyn., E Peppersauce Cave, 32.5249, -110.7106; 11 Jul. 2021; R.W. Mendez leg. – **Santa Cruz Co.** • 4♂, 7♀, 1 imm; Patagonia Mtns, 0.5 mi N Harshaw/Duquesne Road jnct, 31.3917, -110.6885; 6 Nov. 2021; R.W. Mendez, M.A. Leimroth leg.; RWM 21_062. – **Santa Cruz Co.** • 1 imm; Patagonia Mtns, 1 mi E Harshaw Road, 31.4659, -110.7099; 5 Sep. 2021; R.W. Mendez, M.A. Leimroth leg.; RWM 21_035. – **Santa Cruz Co.** • 3♂, 3♀, 1 imm; Patagonia Mtns, 1.5 mi W Harshaw/Duquesne jct., Duquesne Wash, 31.3856, -110.7114; 18 Nov. 2021; R.W. Mendez, C.A. Hamilton leg.; RWM 21_078. – **Santa Cruz Co.** • 1♀, 3 imm; Santa Rita Mtns, Adobe Canyon, 0.5 mi S Bathtub Tank, 31.6730, -110.7601; 16 Sep. 2021; R.W. Mendez leg.; RWM 21_040. – **Santa Cruz Co.** • 1 imm; Santa Rita Mtns, Madera Canyon; date unknown; M. Hedin leg.; MCH 99_010. – **Santa Cruz Co.** • 2♂, 3 imm; Santa Rita Mtns., Aliso Springs, 31.7355, -110.8040; 6 Mar. 2021; R.W. Mendez leg.

***H.apachea* Mule Lineage – Cochise Co.** • ♂, 1 imm; Mule Mtns, 0.5 mi S Mule Pass, Bisbee, 31.4528, -109.9403; 13 Nov. 2021; R.W. Mendez, C.A. Hamilton, M.A. Leimroth leg., RWM 21_073. – **Cochise Co.** • 2♂, 3♀, 3 imm; Mule Mtns, drainage off Escabrosa Ridge, 31.4530, -109.9634; 9 Mar. 2021; R.W. Mendez leg. – **Cochise Co.** • ♂; Mule Mtns, Fissure Peak, 31.4473, -109.9631; 9 Mar. 2021; R.W. Mendez leg.

###### Diagnosis.

**Figure 5.**
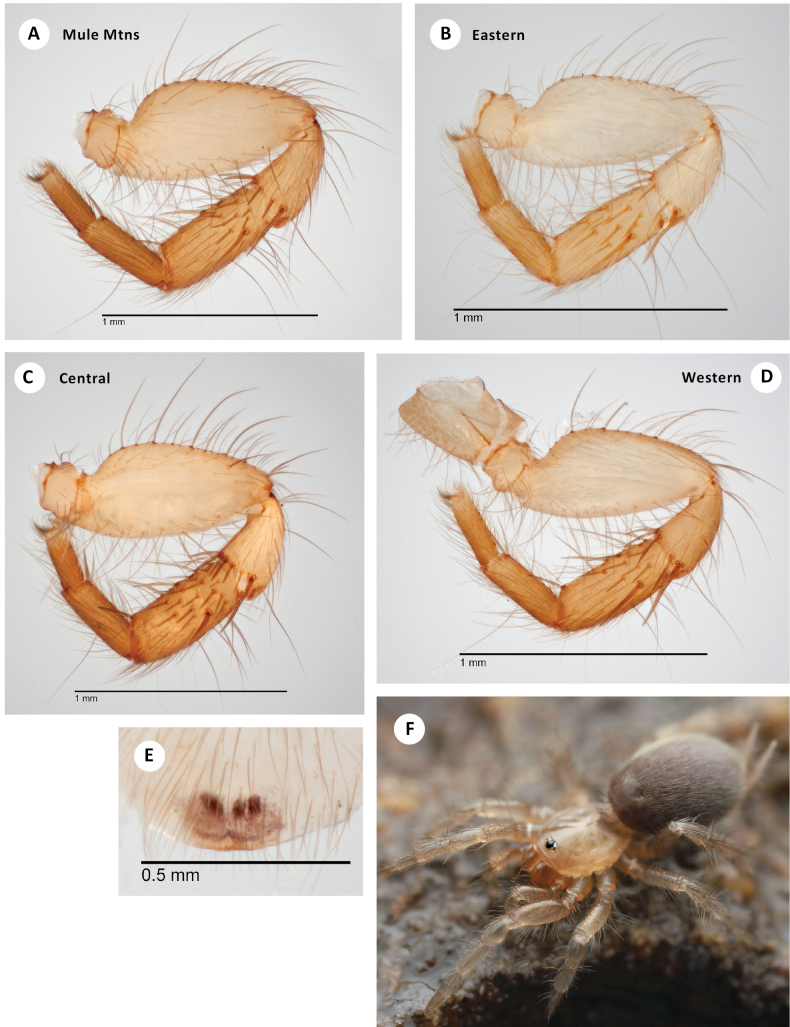
*H.apachea***A** ♂ leg I, prolateral view, Mule Mtns (SDSU_TAC000684) **B** ♂ leg I, prolateral view, Cave Creek Canyon (SDSU_TAC000685) **C** ♂ leg I, prolateral view, Mustang Mtns (SDSU_TAC000686) **D** ♂ leg I, prolateral view, Redington Pass (SDSU_TAC000687) **E** ♀ spermathecae (Mustang Mtns, SDSU_TAC000688) **F** live ♀, Johnny Lyon Hills (RWM 22_009).

This species differs from all other congeners in possessing a diagnostic comb of robust spines distally on the prolateral surface of the male I patella, with a dorsal-most spine that is long and distinctively curved (Fig. 5A–D). The patellar comb is combined with a conspicuous brush of robust prolateral spines on tibia I, again with a dorsal-most spine longer than the others (Fig. 5A–D).

###### Variation.

Representative variation in male leg I patella/tibia spine counts (including some spines tending towards the ventral surface on the patella) is as follows: **Mule Lineage** – Mule Pass (7, 10), Fissure Peak (6, 10); **Western Lineage** – Redington Pass (5, 8), Duquesne Wash (6, 11), Aliso Springs (5, 7); **Central Lineage** – Johnny Lyon Hills (3, 15), French Joe Canyon (6, 12), Mustang Mtns (7, 12); **Eastern Lineage** – Cave Creek Canyon (5, 10), Price Canyon (4, 12), Cochise Trail (5, 9).

###### Distribution.

*Hexurellaapachea* is represented by a series of four phylogeographic lineages distributed north-south through the Cordilleran Gap of southeastern Arizona. COI suggests divergence times between the four clades spanning from the late Miocene to the early Pliocene (Fig. 4); these time estimates are slightly younger (Masta 2000; Derkarabetian et al. 2016) or approximately coincident (Bryson et al. 2013b) with sky island divergence times for other co-distributed arachnid groups. These estimated times are much earlier than the last glacial maximum, when potentially suitable oak and pinyon-juniper forest connected the sky islands (summarized in Moore et al. 2013). With two exceptions (discussed below), populations from individual sky islands form monophyletic genetic groups (Figs 1, 2, 4), suggesting little movement between ranges.

*Vaejovisvorhiesi* and *Pseudouroctonusapacheanus* group scorpions, often collected with *H.apachea*, were diverging in this area throughout the Miocene (with occasional Pleistocene divergences between geographically adjacent ranges) and were dispersing from south to north and east to west, respectively (Bryson et al. 2013a, b). *Vaejovis* Koch, 1836 exhibits a similar pattern to *H.apachea*; a series of lineages oriented north south, but most extending much further north in *Vaejovis*. The biogeographic origin for *H.apachea* remains unclear, and material from northern Sonora and the gap between *H.apachea* and *H.zas* sp. nov. (Fig. 3) will be needed to clarify this directionality.

A comparison can be made with Yarrow’s Spiny Lizard, *Sceloporusjarroviijarrovii* Cope, 1875 regarding the Central and Eastern Clades in *H.apachea*. *Hexurellaapachea* often live at lower elevations than *S.j.jarrovii*, but both utilize rock outcrops and canyons in Madrean Oak Woodlands. Wiens et al. (2019) find a similar east-west split around 4.5 million years ago in *S.j.jarrovii*, divided by the San Pedro River other than Mule Mountains specimens (which fell sister to their western clade). The San Pedro River can be an important barrier resulting in separate lineages inhabiting the sky islands to either side, again seen in the *Vaejovisvorhiesi* group. The rarely sampled Johnny Lyon Hills (RWM22_009), east of the river, should receive more attention to see where their biogeographic affinities typically lie.

The Santa Rita and Patagonia Mountains are closely adjacent, divided by the headwaters of Sonoita Creek. Oak forest comes very low here, connecting the ranges with suitable or near-suitable *Hexurella* habitat. As a result, it is not surprising that *H.apachea* populations may have had contact between these ranges, although the divergences in the Western Clade are not recent (Fig. 4). Similarly, the Pedregosa Mountains are a subrange of the Chiricahuas. This emphasizes the need to include samples from multiple locations per sky island when studying regional species.

###### Natural history.

**Figure 6.**
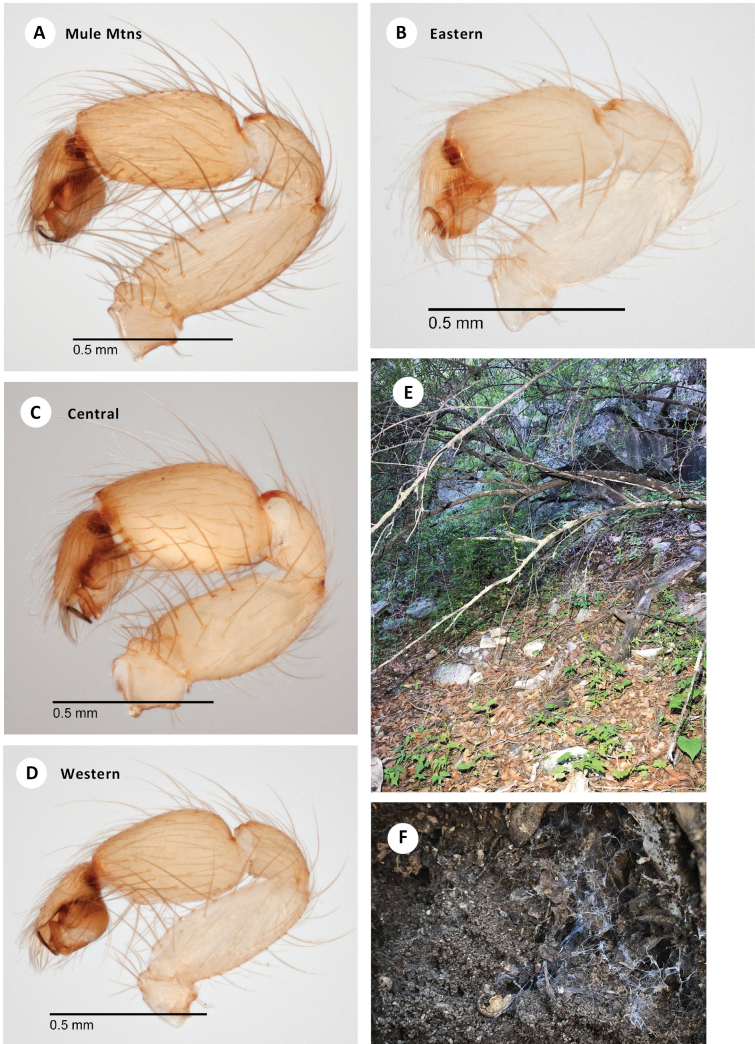
*H.apachea***A** ♂ palp, retrolateral view, Mule Mtns (SDSU_TAC000684) **B** ♂ palp, retrolateral view, Cave Creek Canyon (SDSU_TAC000685) **C** ♂ palp, retrolateral view, Mustang Mtns (SDSU_TAC000686) **D** ♂ palp, retrolateral view, Redington Pass (SDSU_TAC000687) **E** habitat, Whetstone Mtns (RWM 21_029) **F** web, French Joe Canyon (RWM 21_075).

*Hexurellaapachea* is primarily found in low elevation Madrean oak communities between 1400–2075 meters. Nearly all collections have come from flipping small to medium-sized rocks in oak litter (Fig. 6E). Common oak species include *Quercusarizonica* Sarg., *Q.emoryi* Torr., *Q.rugosa* Née, and *Q.toumeyi* Sarg.. Other plants that can also provide suitable litter include the sumacs *Rhustrilobata* Nutt. and *R.virens* Lindh. ex A. Gray, and rarely *Cercocarpusmontanus* Raf. (Mountain Mahogany) or *Celtisreticulata* Torr. (Netleaf Hackberry.) Additionally, *Piptochaetiumfimbriatum* (Kunth) Hitchc. (Pinyon Ricegrass) and *Juniperusdeppeana* Steud. (Alligator Juniper) are useful indicator species, although they are not used in web construction. Aggregations of spiders are usually found along gentle slopes above low riparian corridors or rock outcrops; at the upper end of their elevational range spiders also inhabit north-facing ridgelines.

The litter utilized by *H.apachea* is generally compacted and dense, yet well-draining and without much active fungal activity. Webs are constructed at the transition between litter and soil, consisting of numerous short and interconnected branching tunnels that open into small, space-filling funnel webs wherever voids are present in the leaf matrix (Fig. 6F). *Hexurella* in general are capable of excavating burrows in the top few centimeters of substrate, and often construct a shallow retreat.

Despite their small size, *H.apachea* are typically found in fairly dry microhabitats, especially when compared to the commonly syntopic funnel-web spider *Euagruschisoseus* Gertsch, 1939. In captivity, *H.apachea* has proven to be remarkably desiccation tolerant, requiring no substrate moisture as long as the temperatures remain stable between 20–25 °C and the room has some ambient humidity (10–20%). Like western taxa, *H.apachea* must survive high temperatures and low humidity in the dry season. Egg sacs (laid in late March) have invariably molded when enclosures are kept moist, and the sacs observed in the field are usually placed above the webs, away from the soil in cracks under rocks. In captivity and in the wild, egg sacs are coated with debris, behavior like *E.chisoseus* (RWM, personal observation). Adults likely take two years to mature based on the two overlapping size classes of juveniles usually seen in the wild and captive growth rates, with mature females living for at least two years after maturing.

While patchily distributed throughout their range, *H.apachea* can be dense in appropriate habitats, with 8 males and >75 immatures and females observed (not all collected) in 2 m^2^ in one collection (RWM 21_057) from the type locality in Cave Creek Canyon, Chiricahua Mountains. Small rocks will often have at least one adult female and three or four subadults under them. Despite their proximity in the wild, *H.apachea* (and *Hexurella* in general) do not tolerate cohabitation and readily cannibalize. Mature males have been collected in the field from early October through April; it is unknown if these are overwintering or a different set of males. Males are found running through litter, in 4–5 cm temporary retreats that may function as sperm webs, and the webs of females. Males and juveniles will descend via draglines, permitted by their small size.

###### Discussion.

Because of an overall shared male palpal (Fig. 6A–D) and leg I patella/tibia morphology (see above), we did not explicitly test a multiple species hypothesis for *H.apachea* using genomic algorithmic analyses. However, given the depth of nuclear and mitochondrial divergence and consistently recovered phylogeographic clades (Figs 1, 2, 4), we suspect that such analyses (DELINEATE in particular) would indicate multiple species in this complex. We here favor the more conservative single-species hypothesis, pending additional collecting efforts to the south, north, and east.

###### Conservation status.

Widely distributed and sometimes common in mostly mid-elevation habitats, viewed as secure.

##### ﻿ Hexurella pinea

Taxon classificationAnimaliaAraneaeHexurellidae

Gertsch & Platnick, 1979

30EF2906-52FB-5DD8-8800-349781158EA2

Figs 7 , 8

Hexurella pinea Gertsch & Platnick (1979): 28, figs 70–72, 74, 76–80 (Dmf).

###### Material examined.

**Near-type locality material: USA – Arizona, Yavapai Co.** • 1♂, 1 imm; Brushy Mtn., W of Skull Valley, SW Grasshopper Spring, 34.5555, -112.7475; 13 Apr. 2022; R.W. Mendez leg.; RWM 22_099.

###### Non-type material.

**USA – Arizona, Mohave Co.** • 1♂, 3 imm; Cerbat Mtns, SW Antelope Springs, Antelope Canyon, NE Mt. Tipton, 35.5962, -114.2039; 6 Apr. 2022; R.W. Mendez leg.; RWM 22_087. – **Mohave Co.** • 4♂, 1♀, 3 imm; Music Mtns, NW Garnet Mtn., Fox Canyon, 35.8196, -114.0491; 7 Apr. 2022; R.W. Mendez leg.; RWM 22_088.

###### Diagnosis.

The femur I prolateral surface of male *H.pinea* includes 6–10 larger spines (with a single exception), differing from populations of *H.zas* sp. nov. which possess 11 or more long spines.

###### Variation.

**Figure 7.**
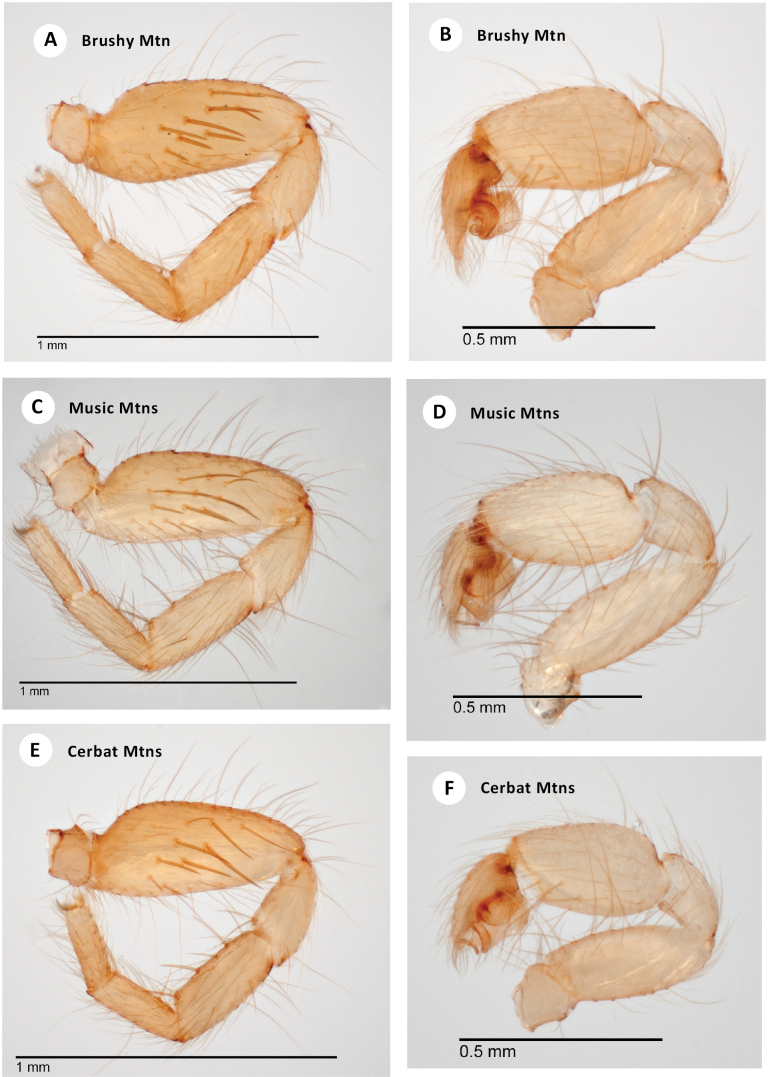
*H.pinea***A** ♂ leg I, prolateral view (SDSU_TAC000691, Brushy Mtn) **B** ♂ palp, retrolateral view (SDSU_TAC000691, Brushy Mtn) **C** ♂ leg I, prolateral view (SDSU_TAC000689, Music Mtns) **D** ♂ palp, retrolateral view (SDSU_TAC000689, Music Mtns) **E** ♂ leg I, prolateral view (SDSU_TAC000690, Cerbat Mtns) **F** ♂ palp, retrolateral view (SDSU_TAC000690, Cerbat Mtns).

Gertsch and Platnick (1979) illustrate the holotype male femur I prolateral surface with ~ 10 larger spines. A male from just west of the type locality at Brushy Mtn (Fig. 3) is similar in condition, possessing nine large spines (Fig. 7A). Males from the more northern Music Mountains possess a femur I prolateral surface with a range of spine numbers (8, 10, 10, 11; Fig. 7C), overlapping the condition found in type or Brushy Mtn samples. The Cerbat Mountains are geographically adjacent to the Musics (Fig. 3), although separated by lower elevation inhospitable habitats. The single male from the Cerbats only has six large femur I prolateral spines (Fig. 7E).

###### Distribution and natural history.

**Figure 8.**
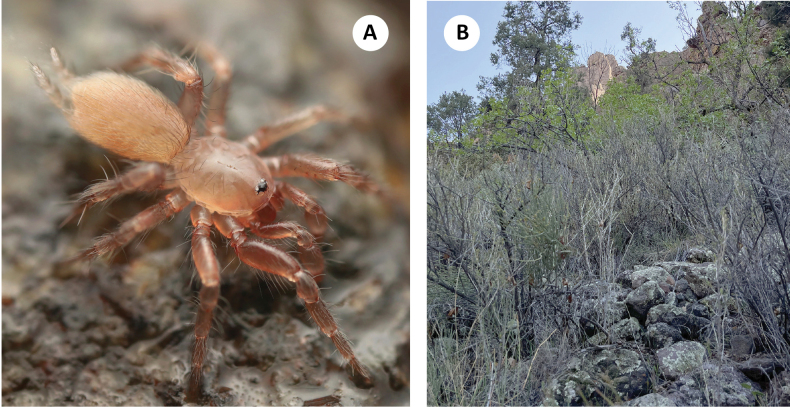
*H.pinea***A** live ♂, Brushy Mtn (RWM 22_099) **B** habitat, Cerbat Mtns (RWM 22_087).

The northernmost known *Hexurella* species, distributed from the Music Mountains on the Colorado River to the Sierra Prieta near Prescott, at elevations between 1400–1575m (Fig. 3). Despite sampling at numerous locales throughout its range, this species has proven to be elusive. The three recent collections have all been from different habitats, and multiple attempts at the type locality have failed to produce specimens. The type series was reportedly collected from “duff of Pinus ponderosa forest (Gertsch and Platnick 1979),” however, we have been unable to recollect them from this kind of litter. At the Brushy Mountain and Music Mountains locales, webs were constructed in nearly completely inorganic granitic gravels under medium to large-sized rocks along slopes. Dried *Quercus* sp. or *Fendlerarupicola* A. Grey (Cliff Fendlerbush) leaves were sometimes incorporated into the webs, but spiders were not found when specifically targeting litter at these locales. The exposed Brushy Mountain locale had sparse vegetation and few trees, while the Music Mountain locale was shaded in a stand of pine-oak forest. At Antelope Springs in the Cerbat Mountains, *H.pinea* was densest (though still uncommon) under stones in a thick patch of *Ephedra* sp. above the spring (Fig. 8B). *Quercus* sp. and *Pteleatrifoliata* L. (Common Hoptree) litter had been blown or washed under many of the rocks.

*Hexurellapinea* males have been the most difficult to collect of the three eastern species. This is likely in part due to the gritty soils seemingly preferred by this species, allowing males not attached to a web to quickly escape into the rapidly collapsing substrate when flipping rocks. At a second locale in the Music Mountains a male was lost because of this; no other individuals were found. Additionally, population densities seem low in *H.pinea*, comparable to the Mojave species *H.ephedra* sp. nov. and *H.xerica* sp. nov. The reduced monsoonal rainfall and cooler winter temperatures throughout the distribution of this species may play a role in keeping populations smaller in this species.

###### Discussion.

DELINEATE and SPEEDEMON *ε* = 0.0185 analyses (Table 2) recover Cerbats+Musics (North) as a separate species from Brushy Mtn (Central). We here conservatively treat these as conspecific based on overlapping patterns of male femur I spination (Fig. 7).

The type locality for *H.pinea*, “5 mi. west of Prescott” (Gertsch and Platnick 1979), is in the Sierra Prieta (Fig. 3). We have not examined type specimens and have been unable to re-collect specimens from the type locality. The Sierra Prieta range is bordered by the Santa Maria Mountains (including Brushy Mountain) to the west, but habitat here is not perceived as contiguous, with the intervening lower elevation Skull Valley. The Sierra Prieta is bordered by the Bradshaw Mountains to the south, and seemingly connected by *Hexurella*-appropriate habitat. More sampling in the Sierra Prieta to Bradshaw Mountains region may find a contact zone between *H.pinea* and *H.zas* sp. nov.

Suitable habitat sampled west of the Colorado River in southern Nevada and the Virgin Mountains failed to produce *Hexurella*. However, due to the often-patchy distribution of this genus and the local scarcity of *H.pinea* (or perhaps other undescribed *Hexurella* species), more collecting is needed in this area.

###### Conservation status.

Likely secure in appropriate habitats, although uncommon.

##### ﻿ Hexurella zas sp. nov.

Taxon classificationAnimaliaAraneaeHexurellidae

FEDF6AA2-A07B-5423-BB1D-F8A63976A444

https://zoobank.org/FC1E1237-52F5-44FA-A718-6B5FCB7DFC96

Fig. 9

Hexurella pinea Hedin et al 2019: figs 3, 4, (in part).

###### Material examined.

**Type material: *Holotype*: – Maricopa Co.** • ♂ holotype; Mt. Ord, 0.5 mi NW Mt. Ord summit, FDR-1688, 33.9125, -111.4145; 15 Apr. 2022; R.W. Mendez leg.; RWM 22_102; SDSU_TAC000693; ***Paratype***: • ♀ paratype; data as for holotype; SDSU_TAC000694.

###### Non-type material.

**USA – Arizona, Maricopa Co.** • 6♀, 5 imm; Mt. Ord, 0.5 mi NW Mt. Ord summit, FDR-1688, 33.9119, -111.4146; 11 Dec. 2021; R.W. Mendez, M.A. Leimroth leg.; RWM 21_082. – **Maricopa Co.** • 9♂; Mt. Ord, 0.5 mi NW Mt. Ord summit, FDR-1688, 33.9125, -111.4145; 15 Apr. 2022; R.W. Mendez leg.; RWM 22_102. – **Yavapai Co.** • 5♂, 3♀, 2 imm; Bradshaw Mtns, 2.15 mi S The Cements, off W Wagoner Road, 34.1374, -112.4475; 14 Apr. 2022; R.W. Mendez leg.; RWM 22_100. – **Yavapai Co.** • 1♀, 1 imm; Bradshaw Mtns, Crown King Road, near Perkins Tunnel Spring, 34.2263, -112.3092; 24 Mar. 2012; M. Hedin, A. Schönhofer, C. Richart, A. DiDomenico, E. Stiner, K. Emata, E. Garcia, D. Sitzmann leg.; MCH 12_009.

###### Diagnosis.

Differs from *H.pinea* in the condition of prolateral male femur I, with *H.zas* sp. nov. possessing 11 or more long spines, totaling a larger number than found in *H.pinea* males (single exception noted above).

###### Description of ♂ holotype

**Figure 9.**
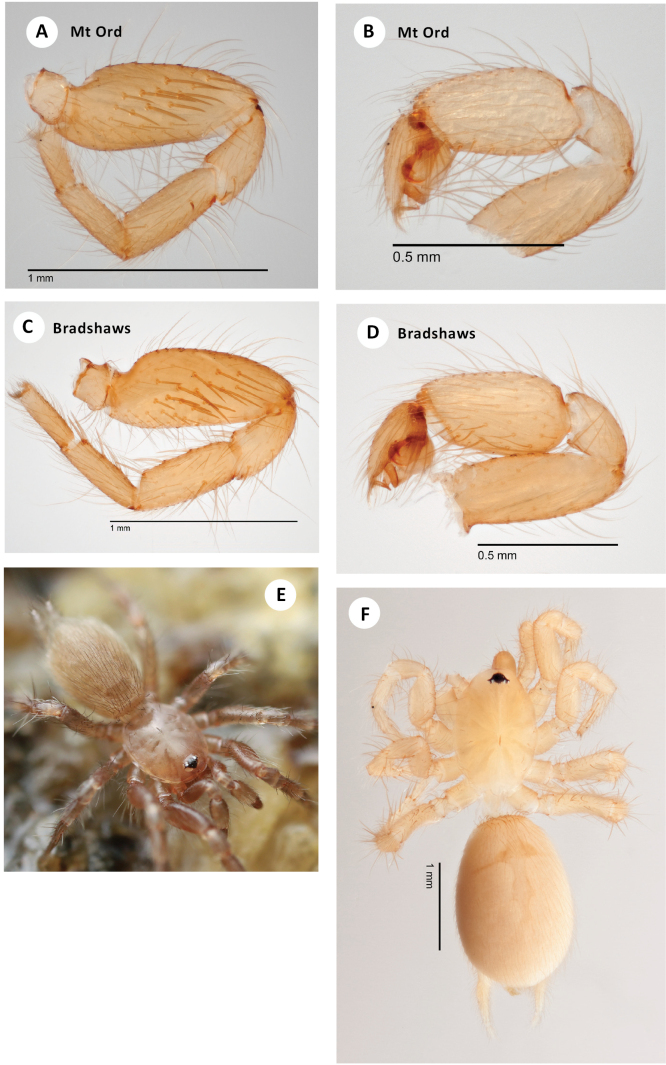
*H.zas* sp. nov. **A** ♂ holotype leg I, prolateral view (SDSU_TAC000693, Mt Ord) **B** ♂ holotype palp, retrolateral view (SDSU_TAC000693, Mt Ord) **C** ♂ leg I, prolateral view (SDSU_TAC000692, Bradshaw Mtns) **D** ♂ palp, retrolateral view (SDSU_TAC000692, Bradshaw Mtns) **E** live ♂, Mt. Ord (RWM 22_102) **F** ♀ paratype (SDSU_TAC000694, Mt Ord).

(SDSU_TAC000693; Fig. 9A–E). Total length (including chelicerae) 2.6, cephalothorax and appendages pale yellow cream (in alcohol), eye tubercle with dark pigmentation beneath. Fangs yellow cream like cephalothorax, with longer basal to medial hairs projecting inwards. Abdomen about concolorous with cephalothorax, evenly covered with fine hairs. Tergal plates barely lighter than abdomen, anterior rectangular plate covering most of abdominal width, posterior oval plate (hard to discern) covering ~ 2/3 abdominal width, both plates covered with fine hairs. Carapace (including chelicerae) 1.125 long, 0.825 wide, suboval in shape as viewed dorsally, gently rounded in front, slightly indented behind. Low and convex viewed laterally, essentially lacking hairs, a few fine hairs towards lateral posterior margins, without evident cephalic grooves, dorsal pigmentation (in alcohol) mostly lacking. Thoracic groove very shallow, linear, barely pigmented, 0.05. Eyes set on low tubercle, ~ 1/3 width of anterior carapace, offset from anterior carapace edge by distance equal to depth of tubercle itself. Anterior lateral eyes 2–3× larger than all others, themselves ca. equal in size. Anterior eye row very slightly procurved, posterior eye row approximately straight. Sternum 0.6 long, 0.5 wide, sparsely covered with hairs concentrated on lateral edges, sternal sigilla not obvious. Labium 0.1 long, 0.2 wide, with forwards-projecting hairs. Endites 0.25 long, 0.2 wide, whitish and thickened medially, forward projecting hairbrushes on prolateral edge. Chelicerae 0.4 long, 0.1 wide at base (viewed from above), promargin with four large teeth, microteeth between; retromargin with one basal microtooth. Leg formula 4132. All legs clothed with fine hairs; legs III and IV with more numerous spines on all surfaces, and with conspicuous spines distally. Leg I thickened, with femur 1/3 as deep as long, prolateral surface of femur with medial patch of 11 spines appearing as two diagonal rows (5 in basal row, 6 in distal row; Fig. 9A), tibia and metatarsus with three and one ventral spines, respectively. Leg I (prolateral view) total length 2.2 (0.75, 0.4, 0.5, 0.4, 0.3). Palp (prolateral view) total length 1.4 (0.5, 0.2, 0.4, 0.3). Palp clothed with fine pale hairs and weak spines; tibia thick, cylindrical, two times as long as deep; weak comb of 3–4 thicker retromarginal hairs on distal edge. Abdomen 1.5 long, 0.9 wide, suboval, somewhat flattened. Posterior median spinnerets slightly shorter than anterior laterals, posterior lateral spinnerets tapering, four-segmented. Embolus closely appressed to the conductor (viewed at 10X magnification).

###### Description of ♀ paratype

(SDSU_TAC000694; Fig. 9F). Total length (including chelicerae) 3.10, cephalothorax and appendages pale cream (in alcohol), including legs. Eye tubercle with dark pigmentation beneath. Fangs pale cream, clothed with long, basal hairs projecting inwards. Abdomen very slightly darker than cephalothorax, densely covered with fine hairs, heart mark apparent. Tergal plates ca. same color but shinier than abdomen, anterior oval plate covering most of abdominal width, posterior oval plate covering ~ 1/3 of abdominal width, both plates covered with fine hairs. Carapace (including chelicerae) 1.27 long, 0.87 wide, suboval in shape as viewed dorsally, gently rounded in front, slightly invaginated behind. Low and convex viewed laterally, essentially lacking hairs, a few fine hairs towards lateral posterior margins, without evident cephalic grooves, dorsal pigmentation (in alcohol) mostly lacking. Thoracic groove shallow, linear, slightly pigmented, 0.125. Eyes set on low tubercle, ~ 1/3 width of anterior carapace, offset from anterior carapace edge by distance equal to depth of tubercle itself. Anterior lateral eyes ~ 3× larger than all others, themselves ca. in size. Anterior eye row very procurved, posterior eye row approximately straight. Sternum 0.7 long, 0.6 wide, sparsely covered with long hairs, sternal sigilla not obvious. Labium 0.1 long, 0.2 wide, gently rounded along whitish anterior edge, with forwards-projecting hairs. Endites 0.375 long, 0.3 wide, whitish and thickened medially, conspicuous forward projecting hairbrushes on prolateral edge. Chelicerae 0.5 long, 0.2 wide at base (viewed from above), promargin with three large teeth, microteeth between; retromargin with one basal microtooth. Leg formula 4132. All legs clothed with fine hairs; legs I and II mostly without dorsal or lateral spines but with ventral spines on tibia and metatarsus; legs III and IV with more numerous spines on all surfaces, and with conspicuous spines distally. Paired tarsal claws with 5–7 microteeth. Leg I (prolateral view) total length 2.2 (0.75, 0.4, 0.4, 0.4, 0.3). Palp (prolateral view) total length 1.5 (0.5, 0.3, 0.4, 0.3), clothed with long hairs, three spines on ventral tibia. Abdomen 1.8 long, 1.2 wide, suboval, somewhat flattened. Posterior median spinnerets slightly shorter than anterior laterals, posterior lateral spinnerets tapering, four-segmented, third segment slightly longer than others and pseudo-segmented. Spermathecae damaged during dissection.

###### Variation.

Important male variation exists in both populations considered. A randomly chosen subset of three non-type males from the type locality (Mt. Ord) reveals a variable number of prolateral femoral spines (11–13), although not arranged in distinct diagonal rows as in the holotype specimen. These males also reveal variation in leg I metatarsus ventral spination, with up to 3 or 4 total spines. Consideration of a randomly chosen subset of four males from the Bradshaw Mtns (RWM 22_100) also reveals a variable number of prolateral femur I spines (from 11–16 long spines, Fig. 9C, D), again not obviously arranged in distinct rows, and with a variable number (3–4) of ventral spines on metatarsus I.

###### Distribution and natural history.

Known from three locations in low to mid-elevation (1325–1850m) habitats in the Arizona transition zone north of Phoenix. Collections in the Bradshaw Mountains are from sclerophyllous oak litter like that preferred by *H.apachea*, while Mt. Ord specimens were taken from nearly pure *Rhamnusilicifolia* Kellogg (Hollyleaf Redberry). The lower elevation collections in the Bradshaws have been in sheltered canyons and rock outcrops, where they are protected from the sun though remain mostly dry, while the higher elevation Mt. Ord locale was on an open, north-facing slope.

Like *H.apachea*, webs consist of a convoluted structure of reticulate tunnels and void-filling sheets. Seemingly less reliant on organic substrates, however they have been collected in crumbly soils and gravel mixtures a short distance away from the main patches of litter. Mature males have been collected in April and May. Dedicated searching at Mt. Ord in mid-December 2021 produced numerous females and immatures, but no males. Pockets of snow were present on the ground, but spiders were still active in their webs. When revisiting the Mt. Ord location the following spring, densities in *H.zas* were similar to *H.apachea*, with multiple individuals sharing small rocks and eleven males seen (two escaping) in an area of approximately 3 m^2^.

###### Etymology.

A noun in apposition which means “snow” in the Western Apache language (Bray, 1998), referencing the colder temperatures and increased snowfall faced by this species in winter. The Western Apache, along with the Yavapai, are the original occupants of the land *H.zas* sp. nov. is found on and their language is undergoing important revitalization efforts.

###### Discussion.

DELINEATE and SPEEDEMON *ε* = 0.0185 analyses (Table 2) recover Bradshaws (South) as a separate species from Mt. Ord (FarSouth). We here conservatively treat these as conspecific, based on overall shared male leg I morphology (Fig. 9), the fact that this morphology varies within sample locations, and that patterns of character variation among these disjunct locations overlap. We recognize that these populations are geographically disjunct with mostly unsuitable intervening habitats (Fig. 3); further sampling in the gap that separates these populations will be important in future research.

###### Conservation status.

Likely secure in appropriate habitats.

#### Western lineage

Well-supported phylogenomic clade of five species (Figs 1, 2, 4), currently known from west of the Colorado River. Includes a nested subclade of three taxa (*H.rupicola*, *H.encina*, *H.uwiiltil* sp. nov.) with contrasting markings on the carapace and overall darker habitus for adults of both sexes (Fig. 1 inset).

**Included species.***Hexurellaephedra* sp. nov., *Hexurellaxerica* sp. nov., *Hexurellarupicola* Gertsch & Platnick, 1979, *Hexurellaencina* Gertsch & Platnick, 1979, *Hexurellauwiiltil* sp. nov.

##### ﻿ Hexurella ephedra sp. nov.

Taxon classificationAnimaliaAraneaeHexurellidae

3FC64F23-605A-5824-B74D-AB6A78B0602E

https://zoobank.org/C4A3A049-E049-4D0D-AAB6-B9439F565876

Fig. 10

###### Material examined.

**Type material: *Holotype***: USA – **California, San Bernardino Co.** • ♂ holotype; Granite Mountains, Deadman’s Hills, 0.5 mi behind Amaral Spring, 34.5148, -117.0640; 17 Feb. 2022; R.W. Mendez leg.; RWM 22_018. ***Paratype***: – **San Bernardino Co.** • ♀ paratype; Granite Mountains, Deadman’s Hills, above Quail Spring, 34.5367, -117.0821; 4 Apr. 2023; R.W. Mendez leg.; RWM 23_032. **Non-type material**: – **San Bernardino Co.** • 3 imm; Granite Mountains, Deadman’s Hills, 0.5 mi behind Amaral Spring, 34.5148, -117.0640; 17 Feb. 2022; R.W. Mendez leg.; RWM 22_018. • 2♂, 1♀, 6 imm; Granite Mountains, Deadman’s Hills, above Quail Spring, 34.5367, -117.0821; 4 Apr. 2023; R.W. Mendez leg.; RWM 23_032.

###### Diagnosis.

**Figure 10.**
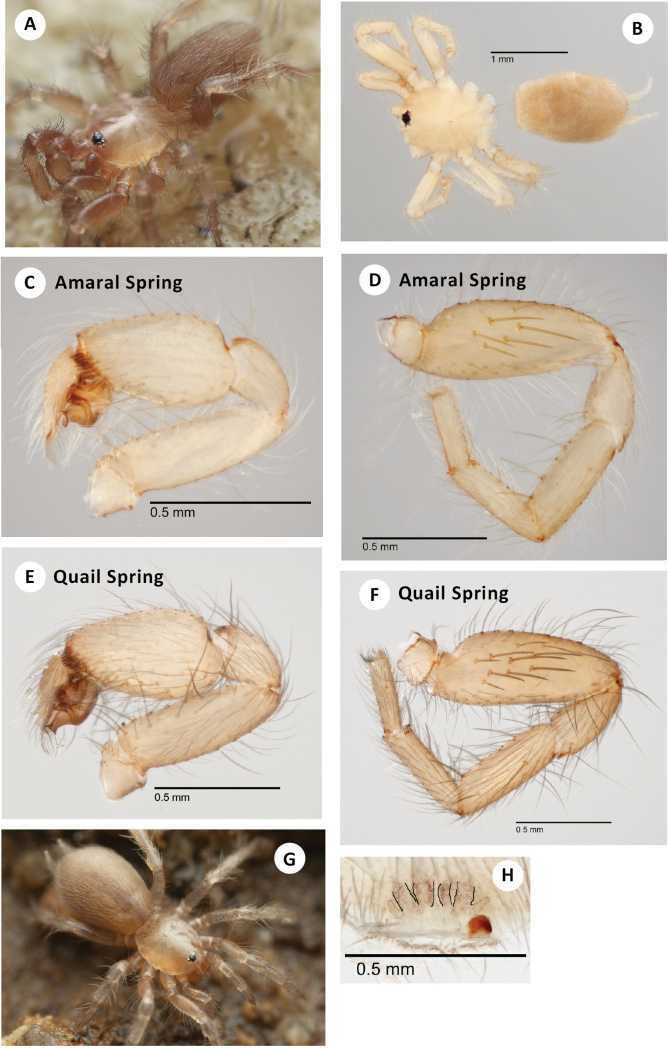
*H.ephedra* sp. nov. **A** live ♂ holotype (SDSU_TAC000680) **B** ♂ holotype (SDSU_TAC000680), dorsal view **C** ♂ palp, retrolateral view, holotype (SDSU_TAC000680) **D** ♂ leg I, prolateral view, holotype (SDSU_TAC000680) **E** ♂ palp, retrolateral view (Quail Spring, RWM 23_032) **F** ♂ leg I, prolateral view (Quail Spring, RWM 23_032) **G** live ♀ (Quail Spring, RWM 23_032) **H** ♀ paratype spermathecae (pencil outline included to show boundaries of medial and lateral receptacles).

Easily distinguished from sister taxon *H.xerica* sp. nov. in that the male palpal tibia possesses a comb of 9 thick distal, retromarginal spines (Fig. 10C, E), a condition unique for the genus. Also, the prolateral surface of male femur I includes a medial patch of 6–10 spines (Fig. 10D, F).

###### Description of ♂ holotype

(TAC_000680; Fig. 10A–D). Total length (including chelicerae) 2.3, cephalothorax and appendages pale cream (in alcohol), eye tubercle with dark pigmentation beneath. Fangs cream colored like cephalothorax, with long, basal to medial hairs projecting inwards. Abdomen slightly darker than cephalothorax, evenly covered with fine hairs. Tergal plates barely lighter than abdomen, anterior rectangular plate covering most of abdominal width, posterior oval plate (difficult to discern) covering ~ 2/3 abdominal width, both plates covered with fine hairs. Carapace (including chelicerae) 1 long, 0.75 wide, sub oval to circular in shape as viewed dorsally, gently rounded in front, slightly indented behind. Low and convex viewed laterally, very sparse fine hairs on lateral posterior margins, without evident cephalic grooves, dorsal pigmentation (in alcohol) mostly lacking. Thoracic groove very shallow, linear, barely pigmented, 0.05. Eyes set on low tubercle, ~ 1/3 width of anterior carapace, offset from anterior carapace edge by distance equal to length of tubercle itself. Anterior lateral eyes ~ 2× as large as others, themselves ca. equal in size. Anterior eye row procurved, posterior eye row approximately straight. Sternum 0.6 long, 0.5 wide, sparsely covered with hairs concentrated on lateral edges, sternal sigilla not obvious. Labium 0.1 long, 0.2 wide, with forwards-projecting hairs. Endites 0.225 long, 0.2 wide, whitish, and thickened medially, hairbrushes projecting forwards on prolateral edge. Chelicerae 0.3 long, 0.1 wide at base (viewed from above), promargin with four large teeth, microteeth between; retromargin with one basal microtooth. Leg formula 4132. All legs clothed with fine hairs, legs III and IV with more numerous spines on all surfaces, and with conspicuous spines distally. Leg I thickened, with femur 1/3 as deep as long, prolateral surface of femur with medial patch of 6 spines (Fig. 10A), tibia and metatarsus with three and two ventral spines, respectively. Leg I (prolateral view) total length 2.2 (0.7, 0.3, 0.5, 0.4, 0.3). Palp (prolateral view) total length 1.4 (0.5, 0.2, 0.5,, 0.3). Palp clothed with fine pale hairs and weak spines; tibia thick, cylindrical, two times as long as deep, comb of nine thicker retromarginal spines on distal edge (Fig. 10B). Abdomen 1.3 long, 0.8 wide, suboval, somewhat flattened. Posterior median spinnerets slightly shorter than anterior laterals, posterior lateral spinnerets tapering and four-segmented. Embolus closely appressed to the conductor (viewed at 10X magnification).

###### Description of ♀ paratype

(SDSU_TAC000695; Fig. 10H). Total length (including chelicerae) 4.8, cephalothorax and appendages dirty cream (in alcohol), including legs. Eye tubercle with dark pigmentation beneath. Fangs pale cream, clothed with long, basal hairs projecting inwards. Abdomen very slightly darker than cephalothorax, densely covered with fine hairs. Tergal plates ca. same color but shinier than abdomen, anterior oval plate covering most of abdominal width, posterior oval plate covering ~ 1/3 of abdominal width, both plates with fine hairs. Carapace (including chelicerae) 2.07 long, 1.30 wide, suboval in shape as viewed dorsally, gently rounded in front, slightly invaginated behind. Low and convex viewed laterally; mostly without hairs, a few fine hairs towards lateral middle and posterior margins, without evident cephalic grooves, dorsal pigmentation (in alcohol) mostly lacking. Thoracic groove shallow, linear, slightly pigmented, 0.125. Eyes set on low tubercle, ~ 1/3 width of anterior carapace, offset from anterior carapace edge by distance equal to depth of tubercle itself. Anterior lateral eyes ~ 3× larger than all others, themselves ca. equal in size. Anterior eye row procurved, posterior eye row approximately straight. Sternum 0.8 long, 0.6 wide, sparsely covered with long hairs, sternal sigilla not obvious. Labium 0.1 long, 0.2 wide, gently rounded along whitish anterior edge, with forwards-projecting hairs. Endites 0.325 long, 0.3 wide, whitish and thickened medially, conspicuous forward-projecting hairbrushes on prolateral edge. Chelicerae 0.5 long, 0.3 wide at base (viewed from above), promargin with three large teeth, microteeth between; retromargin with one basal larger tooth. Leg formula 4132. All legs clothed with fine hairs; legs I and II mostly without dorsal or lateral spines but with ventral spines on tibia and metatarsus; legs III and IV with more numerous spines on all surfaces, and with conspicuous spines distally. Paired tarsal claws with 5–7 microteeth. Leg I (prolateral view) total length 2.5 (0.825, 0.4, 0.5, 0.5, 0.3). Palp (prolateral view) total length 1.6 (0.6, 0.3, 0.3, 0.4), clothed with long hairs, four weak spines on ventral tibia. Abdomen 2.7 long, 1.7 wide, sub oval, somewhat flattened. Posterior median spinnerets slightly shorter than anterior laterals. Posterior lateral spinnerets tapering, four-segmented, third segment slightly longer than others and pseudo-segmented. Spermathecae with medial and lateral receptacles ca. equal length, apparently open-ended; small out-pocketings lateral to receptacles blunt-tipped (Fig. 10H).

###### Variation.

Males from Quail Spring have more femur I spines than topotypic males (Fig. 10D, F), but possess a similar retrolateral palpal comb.

###### Distribution and natural history.

Known only from the Deadman Hills in the Mojave Desert of southern San Bernardino County, California (Fig. 3). Both Amaral and Quail Springs are situated below shallow canyons of rounded, coarse-grain, granitic formations. The plant community on the slopes where the spiders have been found consists mostly of an *Ephedra* sp. (likely *E.viridis* Coville.), *Amsinckia* Lemh. (Fiddlenecks), and seasonal grasses. *H.ephedra* were primarily collected under very large rocks where erosion washed finer soils away and leaving a matrix of coarse gravel, dried *Ephedra* sticks, and other miscellaneous organic material. Despite multiple hours spent searching at both locales, only a few specimens were recovered, likely due to the paucity of accessible habitat and instability of the gravel substrate. The remains of a few webs were observed, with a typical *Hexurella* branching structure. At the type locality small and medium-sized stones revealed no spiders, with only the largest movable rocks having *H.ephedra*. This area has extremely hot and dry summers and the inhabited area would be in full sun for much of the day. *H.ephedra* were more successfully targeted above Quail Spring by focusing on rubble piles along the northern faces of the large granitic outcrops. In the most protected corners *H.ephedra* were flipped under even small stones but were still uncommon. Both adult males from this location were found in female webs.

###### Etymology.

The name is a noun in apposition referring to the gymnosperm genus *Ephedra* L. which was found in close association with the species at the two known localities.

###### Discussion.

Based on shared palpal morphology, seemingly contiguous habitat, and close geographic proximity (~ 2.5 km distant, Fig. 3), we hypothesize that the Quail Spring population represents *H.ephedra* sp. nov. DNA evidence should be collected to further test this hypothesis.

###### Conservation status.

Currently known only from a single small mountain range, and therefore of conservation concern. Further surveys are needed to understand the full distribution of this species, including from other canyons and springs in the Granite Mountains.

##### ﻿ Hexurella xerica sp. nov.

Taxon classificationAnimaliaAraneaeHexurellidae

725F6B17-FBE2-5665-8F62-82D20B68358A

https://zoobank.org/592313AF-5D2B-4B5E-9FE4-34710508306C

Figs 11 , 12

###### Material examined.

**Type material: *Holotype***: USA – **California, San Bernardino Co.** • ♂ holotype; Ord Mountains, Ord Mountain, Sweetwater Spring, 34.6918, -116.8235; 14 Feb. 2022; R.W. Mendez leg.; RWM 22_014. ***Paratype***: –**San Bernardino Co.** • ♀ paratype; Stoddard Ridge, Traer Agua Canyon, 34.6716, -116.9962; 7 Apr. 2023; R.W. Mendez leg.; RWM 23_034. **Non-type material**: – **San Bernardino Co.** • 2 imm; Ord Mountains, Ord Mountain, Sweetwater Spring, 34.6918, -116.8235; 14 Feb. 2022; R.W. Mendez leg.; RWM 22_014. – **San Bernardino Co.** • 3♂, 1 imm; Ord Mountains, Ord Mountain, Sweetwater Spring, 34.6918, -116.8235; 8 Apr. 2023; R.W. Mendez leg.; RWM 23_035. –**San Bernardino Co.** • 3♂, 3♀, 1 imm; Stoddard Ridge, Traer Agua Canyon, 34.6716, -116.9962; 7 Apr. 2023; R.W. Mendez leg.; RWM 23_034. –**San Bernardino Co.** • 4♂, 4♀, 4 imm; Stoddard Mtn, below summit, 34.7003, -117.1236; 6 Apr. 2023; R.W. Mendez leg.; RWM 23_033.

###### Diagnosis.

**Figure 11.**
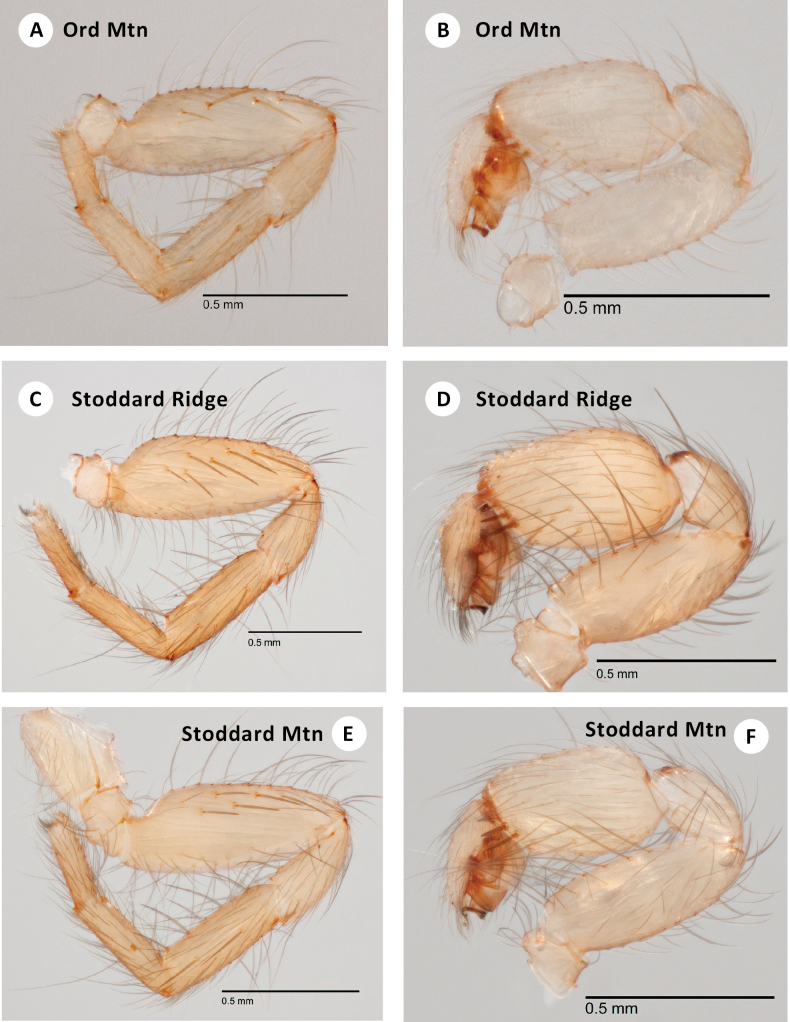
*H.xerica* sp. nov. **A** ♂ leg I, prolateral view, holotype (SDSU_TAC000679) **B** ♂ palp, retrolateral view, holotype (SDSU_TAC000679) **C** ♂ leg I, prolateral view (Stoddard Ridge, RWM 23_034) **D** ♂ palp, retrolateral view (Stoddard Ridge, RWM 23_034) **E** ♂ leg I, prolateral view (Stoddard Mtn, RWM 23_033) **F** ♂ palp, retrolateral view (Stoddard Ridge, RWM 23_033).

Easily distinguished from sister taxon *H.ephedra* in that the male palpal tibia includes only three thick, distal retromarginal spines (Fig. 11B, D F), and male femur I includes a row of 4–7 spines noticeably high on the prolateral face (Fig. 11A, C, E).

###### Description of ♂ holotype

**Figure 12.**
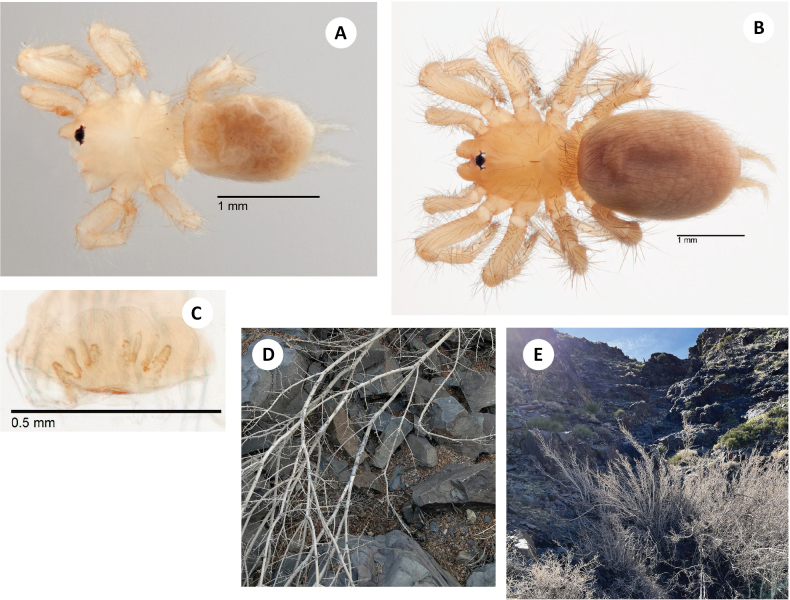
*H.xerica* sp. nov. **A** ♂ holotype (SDSU_TAC000679), dorsal view **B** ♀ paratype (SDSU_TAC000696), dorsal view, **C** ♀ paratype spermathecae **D** type locality (Sweetwater Spring) microhabitat **E** type locality (Sweetwater Spring) macrohabitat.

(SDSU_TAC000679; Fig. 11A, B; Fig. 12A). Total length (including chelicerae) 2.2, cephalothorax and appendages pale cream (in alcohol). Eye tubercle with dark pigmentation beneath. Fangs cream-colored like cephalothorax, with long, basal to medial hairs projecting inwards. Abdomen darker than cephalothorax, hint of circular blotches beneath integument, evenly covered with fine hairs. Tergal plates barely lighter than abdomen, anterior rectangular plate covering most of abdominal width, posterior oval plate covering ~ 2/3 abdominal width, both plates covered with fine hairs. Carapace (including chelicerae) 1.05 long, 0.8 wide, sub oval to circular in shape as viewed dorsally, gently rounded in front, slightly indented behind. Low and convex viewed laterally, very sparse fine hairs on lateral posterior margins, without evident cephalic grooves, dorsal pigmentation (in alcohol) mostly lacking. Thoracic groove very shallow, linear, barely pigmented, 0.075. Eyes set on low tubercle, ~ 1/3 width of anterior carapace, offset from anterior carapace edge by distance equal to length of tubercle itself. Anterior lateral eyes ~ 2× larger than all others, themselves ca. equal in size. Anterior eye row procurved, posterior eye row approximately straight. Sternum 0.5 long, 0.5 wide, sparsely covered with hairs concentrated on lateral edges, sternal sigilla not obvious. Labium 0.1 long, 0.2 wide, with forwards-projecting hairs. Endites 0.225 long, 0.2 wide, whitish, and thickened medially, forward projecting hairbrushes on prolateral edge. Chelicerae 0.3 long, 0.1 wide at base (viewed from above), promargin with five large teeth, microteeth between distalmost pair, retromargin with one basal microtooth. Leg formula 4132. All legs clothed with fine hairs, legs III and IV with more numerous spines on all surfaces, and with conspicuous spines distally. Leg I thickened, with femur 1/3 as deep as long, prolateral surface of femur with dorsal row of 4 spines (Fig. 11A), tibia and metatarsus with three and two ventral spines, respectively. Leg I (prolateral view) total length 2.1 (0.675, 0.4, 0.5, 0.4, 0.3). Palp total length (prolateral view) 1.3 (0.5, 0.2, 0.4, 0.3). Palp clothed with fine pale hairs and weak spines; tibia thick, cylindrical, two times as long as deep, three thicker retromarginal spines on distal edge. Abdomen 1.2 long, 0.8 wide, suboval, somewhat flattened. Posterior median spinnerets slightly shorter than anterior laterals, posterior lateral spinnerets tapering, four-segmented. Embolus closely appressed to the conductor (viewed at 10X magnification).

###### Description of ♀ paratype

(SDSU_TAC000696; Fig. 12B, C). Total length (including chelicerae) 5.00, cephalothorax and appendages pale tan (in alcohol). Eye tubercle with dark pigmentation beneath. Fangs pale cream, clothed with long, basal hairs projecting inwards. Abdomen slightly darker than cephalothorax, evenly covered with fine hairs. Tergal plates ca. same color as abdomen, anterior oval plate covering most of abdominal width, posterior oval plate covering ~ 1/3 of abdominal width, both plates with fine hairs. Carapace (including chelicerae) 2.00 long, 1.50 wide, sub oval in shape as viewed dorsally, gently rounded in front, slightly invaginated behind. Low and convex viewed laterally; mostly without hairs, a few fine hairs along lateral margins, without evident cephalic grooves, dorsal pigmentation (in alcohol) mostly lacking. Thoracic groove shallow, linear, slightly pigmented, 0.125. Eyes set on low tubercle, ~ 1/3 width of anterior carapace, offset from anterior carapace edge by distance equal to depth of tubercle itself. Anterior lateral eyes ~ 3× larger than all others, themselves ca. equal in size. Anterior eye row procurved, posterior eye row approximately straight. Sternum 0.7, long 0.6 wide, sparsely covered with long hairs, sternal sigilla not obvious. Labium 0.1 long, 0.3 wide, gently rounded along whitish anterior edge, with forwards-projecting hairs. Endites 0.35 long, 0.3 wide, whitish and thickened medially, conspicuous forward-projecting hairbrushes on prolateral edge. Chelicerae 0.6 long, 0.3 wide at base (viewed from above), promargin with three large teeth, microteeth between; retromargin with one basal larger tooth. Leg formula 4132. All legs clothed with fine hairs, legs I and II mostly without dorsal or lateral spines but with ventral spines on tibia and metatarsus, legs III and IV with more numerous spines on all surfaces, and with conspicuous spines distally. Paired tarsal claws with 5–7 microteeth. Leg I (prolateral view) total length 2.9 (0.95, 0.5, 0.7, 0.5, 0.4). Palp (prolateral view) total length 1.8 (0.7, 0.3, 0.4, 0.5). Palp clothed with long hairs, four weak spines on ventral tibia. Abdomen 3.0 long, 1.9 wide, sub oval, somewhat flattened. Posterior median spinnerets ca. equal in length to anterior laterals. Posterior lateral spinnerets tapering, four-segmented, third segment slightly longer than others and pseudo-segmented. Spermathecal receptacles appear to be bifurcate, with outer receptacles longer than inner receptacles (Fig. 12C). The inner and outer-most portions are dimpled, suggesting connections to surrounding glands.

###### Variation.

Males from Stoddard Ridge and Stoddard Mountain locations have more femur I spines than topotypic males (Fig. 11C, E), but possess a similar retrolateral palpal comb.

###### Distribution and natural history.

Known only from three adjacent locations in the Mojave Desert of southern San Bernardino County (Fig. 3). The type locality, Sweetwater Spring, is a seep at the bottom of a steep, andesite canyon with a large thicket of *Forestierapubescens* Nutt. (Stretchberry; Fig. 12D, E). The bottom of the ravine consists of a thick layer of litter with a minimal amount of soil over a mixture of smooth, fine-grain gravels of varying sizes. Spiders were found under small-medium sized rocks deep in the thicket, with typical *Hexurella* webs constructed in the gravel and dried leaves. The Stoddard Ridge locale has similar geology, but with numerous, short, winding canyons. Small pockets of spiders were found infrequently under rocks along wash edges, often in *Prunusfasciculata* (Torr.) A. Gray (Desert Almond) litter. The Stoddard Mountain locale was the most exposed, a northeast facing rhyolitic hillside of loosely buried talus with *Ephedra* sp., *Phaceliadistans* Benth. (Desert Scorpionweed), and seasonal grasses. Large, deeply set rocks away from any shelter produced spiders just as well as protected microsites, possibly owing to the deep gravel layer that covers the slopes. This could allow for *H.xerica* to retreat deeper underground in the summer than at other desert *Hexurella* locales.

###### Etymology.

Named to reflect the harsh, xeric conditions in which this species persists, from the Greek *xeros* meaning “dry, withered.”

###### Discussion.

Males from Stoddard Ridge and Stoddard Mountain, which lie ~ 10–15 km west of the type locality, respectively (Fig. 3), differ slightly from topotypic males in femur I spination (Fig. 11A, C, E). Whether or not the habitat between these locations is contiguous is unknown, and DNA evidence should be collected to test our single species hypothesis.

*Hexurellaxerica* sp. nov. populations (Ord Mtn., Stoddard Mtns) are found approximately 50 km north of *Hexurellaephedra* sp. nov. populations (Granite Mtns), possibly separated by low elevation desert habitats of the northern Lucerne Valley (Fig. 3). The morphological and phylogenomic distinctiveness of two previously unknown species in such geographic proximity is surprising. The many dozens of additional isolated mountain ranges in the adjacent Mojave Desert suggests the potential to uncover a radiation of undiscovered, microendemic species in this region.

###### Conservation status.

Currently known only from three adjacent desert locations and therefore of potential conservation concern.

##### ﻿ Hexurella rupicola

Taxon classificationAnimaliaAraneaeHexurellidae

Gertsch & Platnick, 1979

3216A27B-5BF0-50F3-8F35-CF991344D7F7

Fig. 13

Hexurella rupicola Gertsch & Platnick (1979): 31, figs 4, 32, 82, 89–91 (Dmf).
Hexurella rupicola Platnick & Forster (1982): 8, fig. 22.
Hexurella rupicola Hedin et al 2019: figs 1, 3, 4, 5, 6.

###### Material examined.

**Near-type locality material**: USA – **California, Riverside Co.** • ♂, 1 imm; west of Temecula, Rancho California Rd, 33.4973, -117.1694; 22 Dec. 2021; M. Hedin, R. Monjaraz-Ruedas, G. Azevedo leg.; MCH 21_107; **Non-type material**: – **California, San Diego Co.** • ♂; 1.3 mi W of Guatay, Old Hwy 80, N side Guatay Mtn, 32.8541, -116.5754; 26 Jan. 2002; M. Hedin leg.; MCH 02_029; – **San Diego Co.** • ♀, 1 imm; 1.3 mi W of Guatay, Old Hwy 80, N side Guatay Mtn, 32.8541, -116.5754; 24 Mar. 2002; M. Hedin leg.; MCH 02_045; • ♂, 1 imm; 1.3 mi W of Guatay, Old Hwy 80, N side Guatay Mountain, 32.8541, -116.5754; 29 Feb. 2004; M. Hedin leg.; MCH 04_002; • ♀; 1.3 mi W of Guatay, Old Hwy 80, N side Guatay Mountain, 32.8541, -116.5754; 28 Jan. 2006; M. Hedin leg.; MCH 06_010; – **San Diego Co.** • ♂, 2 imm; south side of Viejas Mtn, off I-8 at Williams Rd, 32.8357, -116.7319; 19 Dec. 2021; M. Hedin leg.; MCH 21_105; – **San Diego Co.** • 2♀, 1 imm; near Viejas Mountain trailhead, off Boundary Truck Trail, 32.8550, -116.7400; 11 Mar. 2022; R.W. Mendez leg.; RWM 22_061.– **San Diego Co.** • 2♂, 6♀, 5 imm; San Ysidro Mtns, NW Lupe Spring, off Cottonwood Creek road, 32.5906, -116.7738; 10 Mar. 2022; R.W. Mendez leg.; RWM 22_058.

###### Diagnosis.

**Figure 13.**
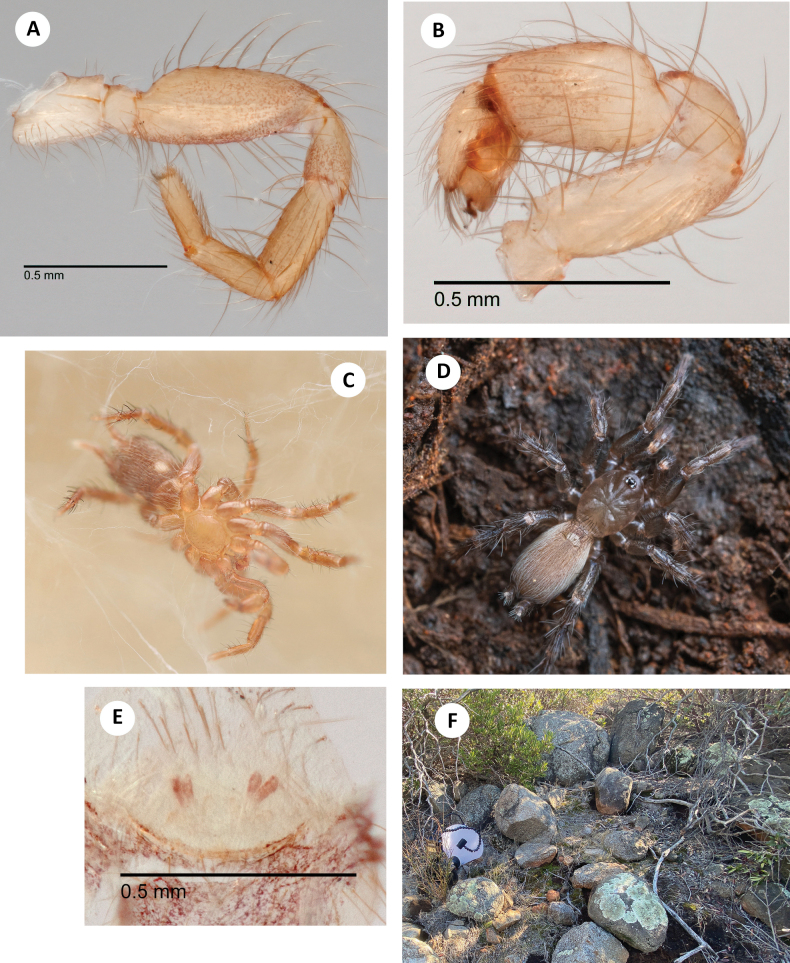
*H.rupicola***A** ♂ leg I, prolateral view (SDSU_TAC000682, San Ysidro Mtns) **B** ♂ palp, retrolateral view (SDSU_TAC000682, San Ysidro Mtns) **C** live ♂ in web (San Ysidro Mtns, RWM 22_058) **D** live ♂ in situ (Viejas Mtn, MCH 21_105), **E** ♀ spermathecae (SDSU_TAC000683, San Ysidro Mtns) **F** Viejas Mtn (MCH 21_105) microhabitat.

Following the original diagnosis of Gertsch and Platnick (1979), *H.rupicola* is similar to *H.encina* in that the prolateral surface of male femur I lacks spines (Fig. 13A), distinguishing these two taxa from all other western clade members. *Hexurellarupicola* differs from *H.encina* in also lacking the distinctive femur I ventral spines found in this latter taxon (Gertsch and Platnick 1979: fig. 86). These authors also note that *H.rupicola* and *H.encina* differ in the separation of the embolus from the conductor, being separated versus closely appressed, respectively (Gertsch and Platnick 1979: figs 87 vs. 90).

###### Variation.

We have examined adult males from three locations in San Diego County (near Guatay, Viejas Mtn, San Ysidro Mtns), south of the type locality in Riverside County. Males from all locations are similar in condition, lacking spines on the prolateral surface of femur I (Fig. 13A).

###### Distribution and natural history.

Known from inland chaparral in Riverside and San Diego Counties. Temecula specimens were uncommonly found under stones in north facing *Adenostomafasciculatum* Hook & Arn. (Chamise) chaparral, while Viejas Mountain specimens were found to be common under well-set intrusive igneous rocks in more exposed, south-facing Chamise chaparral (Fig. 13D, F). The Guatay Mountain collections are north-facing and from slightly higher elevations, with a richer plant community (*Quercus* sp., *A.fasciculatum*, *Arctostaphylos* sp., etc.) but again with intrusive igneous rocks prevailing. Like other western *Hexurella*, *H.rupicola* must deal with long periods of hot and dry conditions in the summer.

###### Discussion.

The gap between *H.rupicola* and the Mojave species requires further sampling. We however note that many devoted mygalomorph biologists have sampled for decades in this region (e.g., Wendell Icenogle in Riverside County) without detecting *Hexurella*.

###### Conservation status.

Likely secure, and likely with a slightly larger distribution than is currently known.

##### ﻿ Hexurella encina

Taxon classificationAnimaliaAraneaeHexurellidae

Gertsch & Platnick, 1979

9B48708C-D75F-51FB-BF48-9F99371100D2

Fig. 14

Hexurella encina Gertsch & Platnick (1979): 30, figs 73, 75, 86–88 (Dm).

###### Material examined.

**Near-type locality material**: MEXICO – **Baja California Norte** • 5♀, 1 imm; Hwy 3, just N Ejido Zapate, N end Guadalupe Valley, 32.1692, -116.5056; 25 Mar. 2022; M. Hedin, R. Monjarez Ruedas, R.W. Mendez leg.; MCH 22_024 • 5♀, 8 imm; Hwy 3, just N Ejido Zapate, N end Guadalupe Valley, 32.1692, -116.5056; 15–16 Jan. 2023; M. Hedin, D. Leavitt leg.; MCH 23_001. **Non-type material**: – **Baja California Norte** • 5♀, 1 imm; road to Cerro Bolla, southeast of Valle de Las Palmas, 32.3300, -116.6454; 25 Mar. 2022; M. Hedin, R. Monjarez Ruedas, R.W. Mendez leg.; MCH 22_023.– **Baja California Norte** • 7♀, 4 imm; road to Sierra San Pedro Martir, W of Hacienda Sinaloa, 30.9815, -116.0960; 28 Mar. 2022; M. Hedin, R. Monjarez Ruedas, R.W. Mendez leg.; MCH 22_037.

###### Diagnosis.

Easily distinguished from sister taxon *H.uwiiltil* sp. nov. in that the *H.encina* male femur I lacks spines on the prolateral surface (Gertsch and Platnick 1979, fig. 86); see *H.rupicola* diagnosis above for differences between *H.encina* and *H.rupicola*.

###### Description of previously undescribed

**Figure 14.**
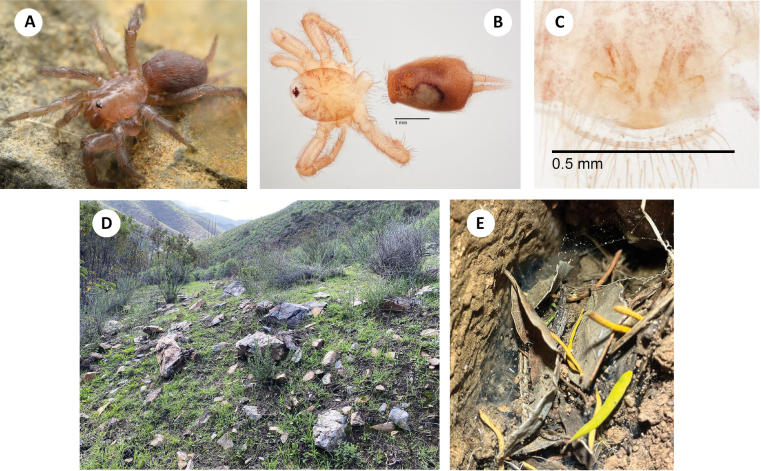
*H.encina***A** ♀ live (NE end Guadalupe Valley, MCH 23_001) **B** ♀ dorsal view (SDSU_TAC000681, NE end Guadalupe Valley) **C** ♀ spermathecae (SDSU_TAC000681, NE end Guadalupe Valley) **D** near type locality habitat (NE end Guadalupe Valley, MCH 23_001), spiders common under rocks along bank **E** web (road to Sierra San Pedro Martir, MCH 22_037).

♀ (SDSU_TAC000681; Fig. 14B, C). Total length (including chelicerae) 4.10, cephalothorax and appendages dirty light brown (in alcohol), legs blotched with pigment. Eye tubercle with dark pigmentation beneath. Fangs concolorous dusky, clothed with long, basal hairs projecting inwards. Abdomen mottled dark purple with whitish background, densely covered with fine hairs. Tergal plates slightly lighter than abdomen, anterior oval plate covering most of abdominal width, posterior oval plate covering ~ 1/3 of abdominal width, both plates covered with fine hairs. Carapace (including chelicerae) 1.87 long, 1.37 wide, suboval in shape as viewed dorsally, gently rounded in front, slightly invaginated behind. Low and convex viewed laterally, inconspicuous fine hairs in ocular region and along carapace edges, without evident cephalic grooves, conspicuous inward-pointed triangular mottled pigmentation above three front leg coxae. Thoracic groove shallow, linear, slightly pigmented,0.2. Eyes set on low tubercle, ~ 1/3 width of anterior carapace, offset from anterior carapace edge by approximately same distance as tubercle length. Anterior lateral eyes ~ 1/3 third larger than all others, themselves ca. equal in size. Anterior eye row procurved, posterior eye row approximately straight. Sternum 0.9 long, 0.8 wide, sparsely covered with long hairs, sternal sigilla not obvious. Labium 0.1 long, 0.4 wide, gently rounded along whitish anterior edge, with forwards-projecting hairs. Endites 0.45 long, 0.4 wide, whitish and thickened medially, conspicuous forward projecting hairbrushes on prolateral edge. Chelicerae 0.7 long, 0.3 wide at base (viewed from above), promargin with three large teeth, microteeth between, retromargin with two basal microteeth. Leg formula 4132. All legs clothed with fine hairs; legs I and II mostly without dorsal or lateral spines but with ventral spines on tibia and metatarsus, legs III and IV with more numerous spines on all surfaces, and with conspicuous spines distally. Paired tarsal claws with 5–7 microteeth. Leg I (prolateral view) total length 3.4 (1.18, 0.6, 0.8, 0.5, 0.4). Palp (prolateral view) total length 2.2 (0.9, 0.4, 0.5,0.6), clothed with long hairs, four spines on ventral tibia. Abdomen 2.2 long, 1.3 wide, suboval, somewhat flattened. Posterior median spinnerets slightly shorter than anterior laterals, posterior lateral spinnerets tapering, four-segmented, third segment slightly longer than others and pseudo-segmented. Spermathecae with medial and lateral receptacles ca. equal length; small pockets lateral to receptacles blunt-tipped (Fig. 14C).

###### ♀ Variation.

Females conspicuously large, with specimens from west of Hacienda Sinaloa (MCH 22_037) the largest females we have seen for this genus.

###### Distribution and natural history.

Spiders at the Ejido Zapate locale were found to be exceedingly common under rubble and small stones in very exposed situations in coastal sage scrub (Fig. 14D). While collections here are from winter and spring months, these microhabitats must be extremely dry in the summer, and we hypothesize that these small spiders retreat into small void spaces deeper in the soil matrix during these times. At all localities *Hexurellaencina* was observed making webs directly out of small voids in clay banks without connecting leaf litter, reminiscent of scaled-down versions of the retreats sometimes created by *Megahexurafulva* in mesic habitats to the north.

The Cerro Bolla and Hacienda Sinaloa collections were from north-facing situations with richer plant communities, and webs were frequently made in a matrix of both leaf litter and millipede frass at the later collection (Fig. 14E). Plants providing shade and litter include *Malosmalaurina* (Nutt.) Nutt. Ex Abrams (Laurel Sumac) and *Rhusintegrifolia* (Nutt.) W.H. Brewer & S. Watson (Lemonade Berry) at Hacienda Sinaloa, and *Quercusagrifolia* Née at Cerro Bolla.

The larger sizes seen in *H.encina* and *H.uwiiltil* sp. nov. may be an adaptation for the exposed microhabitats they inhabit and the increasingly arid conditions moving south into Baja California Norte. The larger size could help with water loss as the surface area/volume ratio shrinks.

###### Discussion.

Gertsch and Platnick (1979) cite the type (and then only known) locality for *H.encina* as “*40 mi. south of Tecate*, *Baja California*, *Norte*”. The main highway from Tecate (Hwy 3) goes approximately straight south, with our Ejido Zapate (N end Guadalupe Valley, MCH 22_024, MCH 23_001) collections being very close to this distance from the border. We presume that these collections represent *H.encina* and describe the previously unknown female from here. Despite collecting attempts at this and several other locations we have not yet collected an adult male *H.encina*.

The southernmost record for this species (west of Hacienda Sinaloa, MCH 22_037) is south of the type locality of *Hexurellauwiiltil* sp. nov. (Fig. 3). Males are unknown for the Hacienda Sinaloa location, and specimens from this location are somewhat phylogenomically divergent from northern locations for *H.encina* (Figs 1, 2, 4). It will be important to collect adult males from this location.

###### Conservation status.

Likely secure, and likely with a larger distribution than currently known.

##### ﻿ Hexurella uwiiltil sp. nov.

Taxon classificationAnimaliaAraneaeHexurellidae

0800911D-ACC1-5116-8EFC-E99596DDC3C9

https://zoobank.org/D57E0E61-DFF8-4ACC-8442-658F181D3086

Figs 15 , 16

###### Material examined.

**Type material: *Holotype***: Mexico – **Baja California Norte** • ♂ holotype; Arroyo Salado, E of Hwy 1, 31.2603, -116.0654; 28 Mar. 2022; M. Hedin, R. Monjarez Ruedas, R.W. Mendez leg.; SDSU_TAC000677. ***Paratype***: • ♀ paratype; data as for holotype; SDSU_TAC000678; **Non-type material**: • 7♀; data as for holotype; MCH 22_041.

###### Diagnosis.

**Figure 15.**
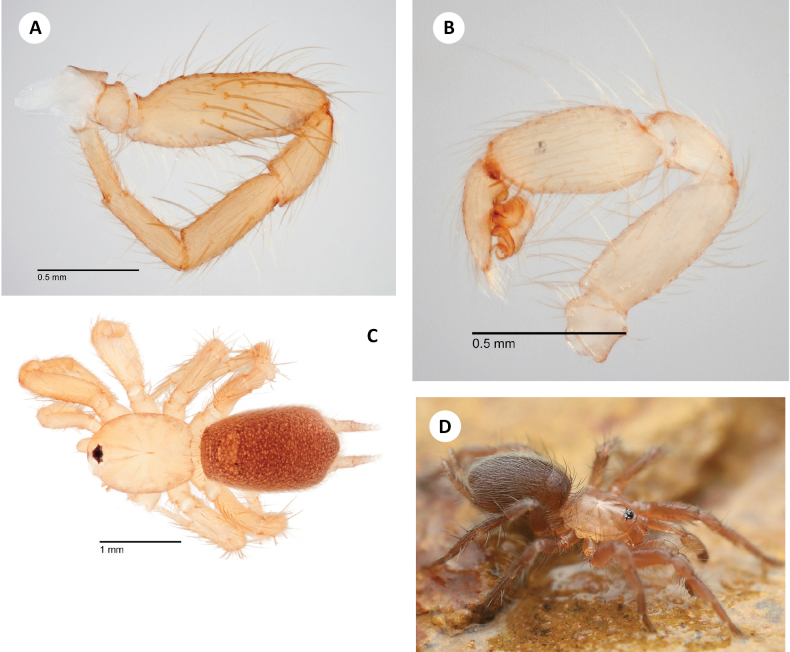
*H.uwiiltil* sp. nov. **A** ♂ leg I, prolateral view, holotype (SDSU_TAC000677) **B** ♂ palp, retrolateral view, holotype (SDSU_TAC000677) **C** ♂ holotype (SDSU_TAC000677) **C** ♂ holotype (SDSU_TAC000677), live.

This species differs from all other members of the western clade in that the male femur I includes a patch of 10 prolateral spines (Fig. 15A), reminiscent of the femur I morphology found in eastern clade members *H.pinea* and *H.zas* sp. nov. (Figs 7, 9).

###### Description of ♂ holotype

(SDSU_TAC000677; Fig. 15). Total length (including chelicerae) 2.7, cephalothorax and appendages pale tan (in alcohol), eye tubercle with dark pigmentation beneath. Fangs colored like cephalothorax, with long, basal hairs projecting inwards. Abdomen mottled dark purple with whitish background, evenly covered with fine hairs, tergal plates barely lighter than abdomen, anterior rectangular plate covering most of abdominal width, posterior oval plate (with posterior indent) covering ~ 2/3 abdominal width, both plates covered with fine hairs. Carapace (including chelicerae) 1.23 long, 0.88 wide, suboval in shape as viewed dorsally, gently rounded in front, slightly indented behind. Carapace low and convex viewed laterally, very sparse fine hairs in ocular region and along carapace edges, without evident cephalic grooves, inward-pointed triangular mottled pigmentation above three front leg coxae. Thoracic groove very shallow, linear, slightly pigmented, 0.1. Eyes set on low tubercle, ~ 1/2 width of anterior carapace, offset from anterior carapace edge by slightly less distance as length of tubercle itself. Anterior lateral eyes ~ 2× as large as others, themselves ca. equal in size. Anterior eye row procurved, posterior eye row approximately straight. Sternum 0.6 long, 0.5 wide, sparsely covered with hairs, more hairs on lateral edges, sternal sigilla not obvious. Labium 0.1 long, 0.2 wide, with forwards-projecting hairs. Endites 0.25 long, 0.3 wide, whitish, and thickened medially, forward projecting hairbrushes on prolateral edge. Chelicerae 0.4 long, 0.1 wide at base (viewed from above), promargin with three large teeth, microteeth between; retromargin with three basal microteeth. Leg formula 4132. All legs clothed with fine hairs; legs III and IV with more numerous spines on all surfaces, and with conspicuous spines distally. Leg I thickened, femur one-third as deep as long, prolateral surface of femur with patch of 10 spines, two spines at femur/patella junction, tibia and metatarsus with three and two ventral spines, respectively (Fig. 15A). Leg I (prolateral view) total length 2.6 (0.875, 0.4, 0.5, 0.5, 0.3). Palp (prolateral view) total length 1.7 (0.5, 0.3, 0.5, 0.4). Palp clothed with fine pale hairs and weak spines. Palpal tibia thick, cylindrical, ~ 2× as long as deep, five thicker distal spines on retromarginal tibia/ tarsus joint. Abdomen 1.5 long, 1.0 wide, suboval, somewhat flattened. Posterior median spinnerets slightly shorter than anterior laterals, posterior lateral spinnerets tapering, four-segmented, third segment slightly longer than others and pseudo-segmented. Embolus closely appressed to the conductor (viewed at 10X magnification).

###### Description of ♀ paratype

**Figure 16.**
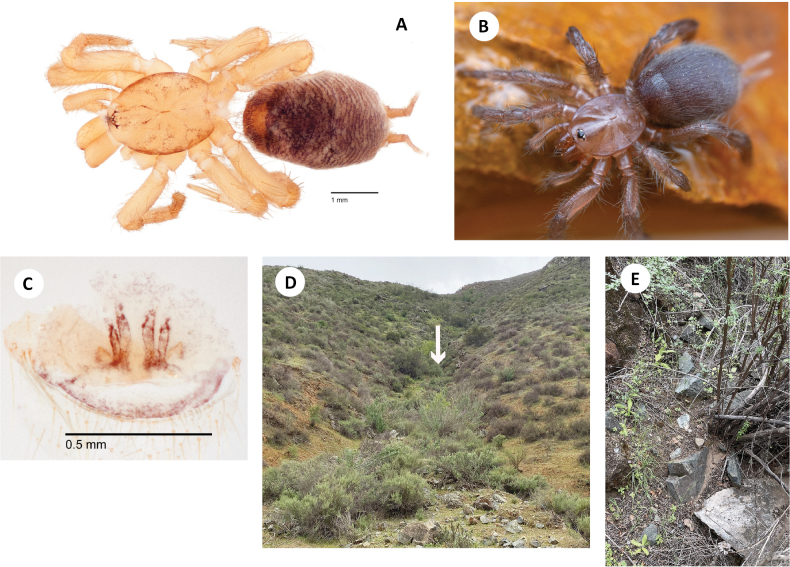
*H.uwiiltil* sp. nov. **A** ♀ paratype (SDSU_TAC000678), dorsal view **B** ♀ paratype (SDSU_TAC000678), live **C** ♀ paratype (SDSU_TAC000678), spermathecae **D** type locality habitat (Arroyo Salado, MCH 22_041), spiders found in small ravine at arrow **E** type locality microhabitat.

(SDSU_TAC000678; Fig. 16A–C). Total length (including chelicerae) 5.0, cephalothorax and appendages dirty light brown (in alcohol), eye tubercle with dark pigmentation beneath. Fangs concolorous dusky, clothed with long, basal hairs projecting inwards. Abdomen mottled dark purple with a whitish background, densely covered with fine hairs. Tergal plates lighter than abdomen, anterior oval plate covering most of abdominal width, posterior oval plate covering ~ ½ of abdominal width, both plates with fine hairs. Carapace including chelicerae 2.2 long, 1.6 wide; suboval in shape as viewed dorsally, gently rounded in front, slightly invaginated behind; low and convex viewed laterally; inconspicuous fine hairs in ocular region and along carapace edges, without evident cephalic grooves, mottled pigmentation above three front leg coxae. Thoracic groove shallow, linear, slightly pigmented, 0.2. Eyes set on low tubercle, ~ 1/3 width of anterior carapace, offset from carapace edge by approximately same distance as tubercle length. Anterior lateral eyes ~ 1/3 larger than all others, themselves ca. equal in size. Anterior eye row procurved, posterior eye row approximately straight. Sternum 1.2 long, 0.9 wide, sparsely covered with long hairs, sternal sigilla not obvious. Labium 0.1 long, 0.4 wide, gently rounded along whitish anterior edge, with forwards-projecting hairs. Endites 0.5 long, 0.4 wide, whitish, and thickened medially, conspicuous forward projecting hairbrushes on prolateral edge. Chelicerae 0.7 long, 0.3 wide at base (viewed from above), promargin with three large teeth, microteeth between and basal to last promarginal macrotooth; retromargin with single basal tooth. Leg formula 4132. All legs clothed with fine hairs, legs I and II mostly without dorsal or lateral spines but with ventral spines on tibia and metatarsus, legs III and IV with more numerous spines on all surfaces, and with conspicuous spines distally. Paired tarsal claws with 5–7 microteeth. Leg I (prolateral view) total length 3.9 (1.3, 0.75, 0.8, 0.5, 0.4). Palp (prolateral view) total length 2.2 (0.9, 0.4, 0.4, 0.5), clothed with long hairs, three spines on ventral tibia. Abdomen 2.8 long, 1.9 wide, suboval, somewhat flattened. Posterior median spinnerets are slightly shorter than anterior laterals. Posterior lateral spinnerets tapering, four-segmented, third segment pseudo-segmented and slightly longer than others. Spermathecae with thin medial receptacles slightly longer than lateral receptacles; small pockets lateral to receptacles with nipple-like extensions (Fig. 16C).

###### Variation.

Only a single male is known. We did not dissect and compare spermathecae from other topotypic females, but these non-paratype specimens are like the paratype in body size and markings (e.g., dark abdomens, dark carapace edges, etc.).

###### Distribution and natural history.

Only known from the type locality, a north-facing ravine in low coastal desert (Fig. 16D, E). Females and immatures were common under small to medium-sized rocks and litter along the slope. Vegetation consisted of abundant annual ground covers with shade and litter coming from tall shrubs growing along the drainage, likely *Rhamnuscrocea* Nutt. (Redberry Buckthorn). The microhabitat was like the Hacienda Sinaloa locale, with dry millipede frass making up a considerable amount of the matrix the webs were constructed in. The litter, frass, and rocks overlaid a gravel mixture the spiders could easily retreat into, and at least one male was lost this way.

###### Etymology.

A noun in apposition which means spider in the Kiliwa language. The Kiliwa are indigenous peoples of northern Baja California, originally inhabiting an area surrounding the Sierra de San Pedro Mártir. The Kiliwa language is in danger of extinction, with fewer than 50 speakers in a recent census. We honor them and their language by naming this species in the same way they named these spiders centuries ago.

###### Discussion.

Both mitogenomic and nuclear phylogenomic data support *Hexurellauwiiltil* sp. nov. as sister to *H.encina* (Figs 1, 2, 4), also endemic to northern Baja California Norte.

###### Conservation status.

This species is currently known only from a single locality, with bounding locations for *H.encina* to the north and south (Fig. 3). More collecting effort is needed to understand the distribution and conservation status of *Hexurellauwiiltil* sp. nov.

## Supplementary Material

XML Treatment for Hexurella

XML Treatment for Hexurella apachea

XML Treatment for Hexurella pinea

XML Treatment for Hexurella zas

XML Treatment for Hexurella ephedra

XML Treatment for Hexurella xerica

XML Treatment for Hexurella rupicola

XML Treatment for Hexurella encina

XML Treatment for Hexurella uwiiltil

## ﻿Additional information

### Author ORCIDs

Rodrigo Monjaraz-Ruedas https://orcid.org/0000-0002-6462-3739

### Data availability

All of the data that support the findings of this study are available in the main text or Supplementary Information.

## Supplementary materials

Supplementary material 1 Molecular and morphological voucher information Data type occurences, morphological, phylogenetic Explanation note Voucher specimen and genomic data information. https://binary.pensoft.net/file/864142This dataset is made available under the Open Database License (http://opendatacommons.org/licenses/odbl/1.0/). The Open Database License (ODbL) is a license agreement intended to allow users to freely share, modify, and use this Dataset while maintaining this same freedom for others, provided that the original source and author(s) are credited.Rodrigo Monjaraz-Ruedas, Raymond Wyatt Mendez, Marshal Hedin

Supplementary material 2 Custom scripts for bioinformatic analyses Data type scripts https://binary.pensoft.net/file/864143This dataset is made available under the Open Database License (http://opendatacommons.org/licenses/odbl/1.0/). The Open Database License (ODbL) is a license agreement intended to allow users to freely share, modify, and use this Dataset while maintaining this same freedom for others, provided that the original source and author(s) are credited.Rodrigo Monjaraz-Ruedas, Raymond Wyatt Mendez, Marshal Hedin

Supplementary material 3 Input matrices, analysis log files, and output tree files for nuclear and mitochondrial analyses Data type data matrices, etc. https://binary.pensoft.net/file/864144This dataset is made available under the Open Database License (http://opendatacommons.org/licenses/odbl/1.0/). The Open Database License (ODbL) is a license agreement intended to allow users to freely share, modify, and use this Dataset while maintaining this same freedom for others, provided that the original source and author(s) are credited.Rodrigo Monjaraz-Ruedas, Raymond Wyatt Mendez, Marshal Hedin

Supplementary material 4 Ingroup + outgroup UCE concatenated ML tree. Specimen numbers correspond to those in Suppl. material 1. Data type phylogenetic tree https://binary.pensoft.net/file/864145This dataset is made available under the Open Database License (http://opendatacommons.org/licenses/odbl/1.0/). The Open Database License (ODbL) is a license agreement intended to allow users to freely share, modify, and use this Dataset while maintaining this same freedom for others, provided that the original source and author(s) are credited.Rodrigo Monjaraz-Ruedas, Raymond Wyatt Mendez, Marshal Hedin

Supplementary material 5 Ingroup-only mitochondrial ML gene tree. Specimen numbers correspond to those in Suppl. material 1. Data type mitochondrial ML gene tree https://binary.pensoft.net/file/864146This dataset is made available under the Open Database License (http://opendatacommons.org/licenses/odbl/1.0/). The Open Database License (ODbL) is a license agreement intended to allow users to freely share, modify, and use this Dataset while maintaining this same freedom for others, provided that the original source and author(s) are credited.Rodrigo Monjaraz-Ruedas, Raymond Wyatt Mendez, Marshal Hedin
