# Supplementary figures and images for: ﻿Species delimitation, biogeography, and natural history of dwarf funnel web spiders (Mygalomorphae, Hexurellidae, Hexurella) from the United States / Mexico borderlands
Source: Zookeys. 2023 Jun 14;1167:109–57. doi: 10.3897/zookeys.1167.103463 (PMC10285686; doi:10.3897/zookeys.1167.103463)

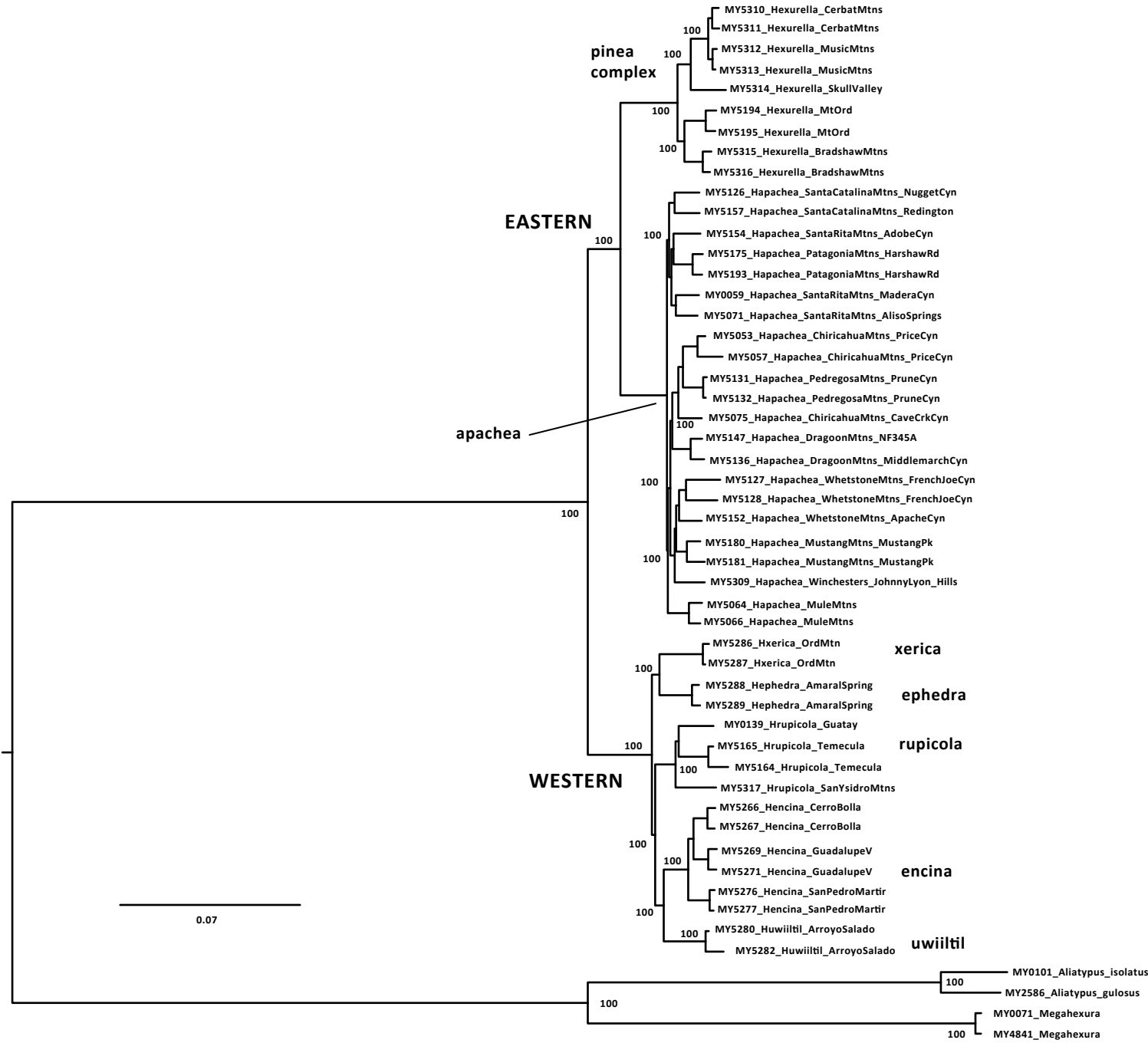

Supplement: Supplementary material 4 — Ingroup + outgroup UCE concatenated ML tree. Specimen numbers correspond to those in Suppl. material 1. [file zookeys-1167-109_article-103463__-s004.pdf]

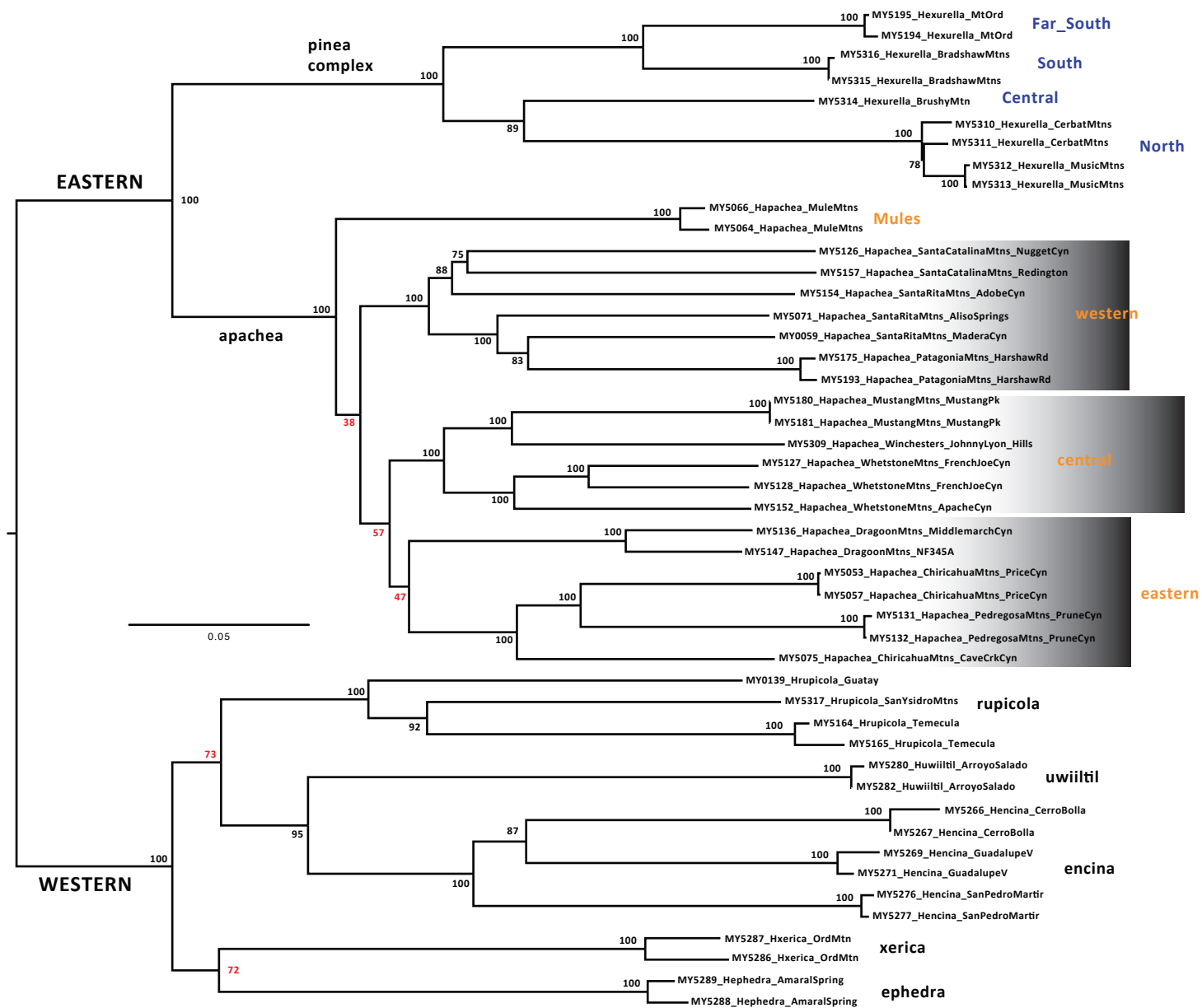

Supplement: Supplementary material 5 — Ingroup-only mitochondrial ML gene tree. Specimen numbers correspond to those in Suppl. material 1. [file zookeys-1167-109_article-103463__-s005.pdf]
